# A systematic review of mathematical models for soil-transmitted helminthiasis control strategies

**DOI:** 10.1371/journal.pntd.0013431

**Published:** 2026-08-28

**Authors:** Lemjini Masandawa, Miracle Amadi, Isambi S. Mbalawata, Safari Kinung’hi, Silas S. Mirau

**Affiliations:** 1 School of Computational and Communication Science and Engineering, The Nelson Mandela African Institution of Science and Technology, Arusha, Tanzania; 2 School of Engineering Science, Lappeenranta University of Technology, Lappeenranta, Finland; 3 African Institute for Mathematical Sciences (AIMS) Research and Innovation Centre (RIC), NEI Global Secretariat, Kigali, Rwanda; 4 National Institute for Medical Research (NIMR), Mwanza Centre, Mwanza, Tanzania; University of Liverpool, UNITED KINGDOM OF GREAT BRITAIN AND NORTHERN IRELAND

## Abstract

**Background:**

Soil-transmitted helminthiasis (STH) remains an important public health challenge in endemic settings, and mathematical transmission models are widely used to evaluate STH transmission and control strategies. However, no systematic review has comprehensively classified STH transmission models according to their biological and structural characteristics and intervention designs while examining the operational incorporation of World Health Organization (WHO) roadmap targets and projected timelines. This review therefore aimed to classify STH transmission models and interventions and examine the operational incorporation of WHO roadmap targets and projected timelines.

**Methodology:**

We searched Web of Science, Scopus, and Embase for English-language studies published between January 2015 and December 2024. Five reviewers participated in screening and data extraction. Models were classified by transmission unit, mathematical framework, and population structure, and interventions by strategy, delivery platform, target population, and treatment regimen. WHO alignment was classified as explicit or implicit against the 2011–2020 and 2021–2030 roadmaps using pre-defined criteria. Projected timelines were extracted, and methodological quality was assessed using the Assessment of Modelling Studies tool.

**Findings:**

Forty-two studies satisfied the inclusion criteria. Parasite-based (69%), age-structured (67%), and deterministic (62%) formulations were frequently represented than host-based (31%), homogeneous (33%), and stochastic (38%) formulations, respectively. Preventive chemotherapy was the most frequently modelled intervention strategy (81%), whereas integrated strategies represented 19%. Thirty-three studies (79%) aligned with at least one WHO roadmap objective, including 12 (29%) explicitly and 21 (50%) implicitly aligned studies. Fourteen studies (33%) reported projected timelines for WHO-relevant outcomes. External validation was reported in 14% of studies.

**Conclusion:**

The literature was predominantly characterised by parasite-based, age-structured, and deterministic formulations, with preventive chemotherapy forming the principal intervention focus. Integrated strategies were less represented. Projected timelines and external validation were also infrequently reported. Models addressing programme planning or policy evaluation could benefit from relevant integrated interventions, projected timelines, and external validation.

## Author summary

Soil-transmitted helminthiases (STH) remain an important public health challenge in endemic settings. The World Health Organization (WHO) has established targets for reducing STH-related morbidity and advancing elimination as a public health problem. Mathematical models are widely used to evaluate STH transmission and control strategies, but their structural characteristics, intervention designs, incorporation of WHO roadmap targets, and projected timelines have not been systematically reviewed. We reviewed 42 mathematical modelling studies published between 2015 and 2024 to classify STH transmission models and interventions and examine the operational incorporation of WHO roadmap targets and reported projected timelines. The reviewed studies were predominantly parasite-based, age-structured, and deterministic. Preventive chemotherapy was the most frequently represented intervention strategy, while integrated strategies were less frequently represented. Most studies aligned with at least one WHO roadmap objective, although implicit alignment was more common than explicit alignment. Only a subset reported projected timelines for WHO-relevant outcomes, while external validation and uncertainty quantification were infrequently reported. Overall, the findings provide a structured overview of current STH modelling approaches and identify less frequently represented areas. Models addressing programme planning or policy evaluation could benefit from relevant integrated interventions, clearly defined WHO targets, projected timelines, validation, and uncertainty assessment.

## Introduction

Soil-transmitted helminthiasis (STH) is among the diseases classified by the World Health Organization (WHO) as neglected tropical diseases (NTDs), reflecting its disproportionate burden in low-resource settings and the relatively limited investment in research [1]. STH comprises infections caused by four major nematode species: *Ascaris lumbricoides*, *Trichuris trichiura*, and the hookworms *Necator americanus* and *Ancylostoma duodenale*, with *Strongyloides stercoralis* increasingly recognized as an additional contributor to the disease burden [1–3]. Globally, more than 1.5 billion people are infected across approximately 166 endemic countries, with an estimated 4.5 billion individuals at risk [4–6]. The associated burden, measured in disability-adjusted life years (DALYs), ranges from 5 to 39 million, indicating substantial impacts on population health [7].

STH disproportionately affects impoverished and marginalized populations, particularly in regions with limited access to safe water, sanitation, and hygiene (WASH), including sub-Saharan Africa, parts of Asia, and Latin America [8]. Chronic and heavy infections are associated with anemia, malnutrition, impaired physical and cognitive development in children, and reduced productivity in adults, thereby reinforcing cycles of poverty and health inequity [9,10].

Global control efforts have been guided by successive WHO roadmaps. The 2011–2020 WHO roadmap focused primarily on morbidity control through preventive chemotherapy (PC), targeting at least 75% treatment coverage among at-risk pre-school-aged children (PSAC) and school-aged children (SAC) [11], with subsequent expansion of treatment programmes to other at-risk groups such as women of reproductive age [12,13]. The 2021–2030 roadmap places greater emphasis on elimination of STH as a public health problem and on integrated approaches that combine PC with complementary measures such as water, sanitation, and hygiene, education, and other behavioural interventions [14,15].

Preventive chemotherapy remains a central component of STH control and involves the periodic administration of anthelmintic drugs such as albendazole or mebendazole to at-risk populations [4,16]. Alternative treatment regimens have also been considered, including dual- or triple-drug therapy, the latter involving three anthelmintic agents, for example albendazole, ivermectin, and pyrantel pamoate [5,6].

Mathematical models have been widely used to examine infectious disease dynamics [17–20]. In the context of STH, these models provide a framework for examining transmission dynamics and evaluating intervention strategies under different epidemiological and programme conditions. Model analyses have considered alternative intervention scenarios, control and elimination outcomes, and projected timelines [21–23].

### Rationale of the study

Given the increasing use of mathematical models to examine STH transmission and control strategies, a systematic characterization of the existing modelling literature is needed. Existing reviews have largely focused on broader aspects of neglected tropical disease (NTD) control [24–28], without specifically synthesizing STH transmission models according to their biological and structural characteristics, the interventions represented within them, the operational incorporation of WHO roadmap targets, and reported projected timelines. Consequently, the distribution of existing STH models across model and intervention characteristics, the ways in which WHO-defined quantitative targets or programme criteria are incorporated into model specification or analysis, and the extent to which projected timelines are reported have not been systematically characterized. A systematic review using a pre-defined protocol and PRISMA-guided methods provides a structured approach for addressing these questions.

This review aimed to: (i) systematically classify STH transmission models according to their biological and structural characteristics; (ii) decompose STH control interventions represented in mathematical models into orthogonal components and systematically classify them using these components; and (iii) examine the operational incorporation of WHO 2011–2020 and 2021–2030 roadmap targets and summarize the projected timelines reported by the included studies.

This review contributes to the STH modelling literature by providing a structured taxonomy of mathematical models according to transmission unit, mathematical framework, and population structure; systematically classifying interventions according to intervention strategy, delivery platform, target-population representation, and treatment regimen; examining the operational incorporation of WHO roadmap targets and reported projected timelines; and characterizing methodological features of the reviewed modelling literature.

The study is guided by the following three interconnected research questions:

- **Model taxonomy:** How are STH transmission models classified according to their biological assumptions, mathematical formulation, and demographic structure?
- **Intervention taxonomy:** How are STH control interventions represented in mathematical models classified according to intervention strategy, delivery platform, target-population representation, and treatment regimen?
- **WHO target alignment and reported timelines:** How are WHO-defined quantitative targets or programme criteria operationally incorporated into STH model specification or analysis, and what projected timelines are reported by the included studies?

## Materials and methods

This study followed established procedures for conducting a systematic review in accordance with the Preferred Reporting Items for Systematic Reviews and Meta-Analyses (PRISMA) 2020 statement [29,30]. The application of PRISMA guidelines was intended to minimize bias throughout the review process and enhance the validity of findings related to mathematical modelling of helminthiasis control strategies. A study protocol was developed, as detailed in the supplementary files (see S5 File). The completed PRISMA 2020 checklist and abstract are provided as Supporting information (see S1 File).

### Information sources and search terms

A comprehensive literature search was conducted for peer-reviewed articles published between January 2015 and December 2024 in three bibliographic databases: Web of Science, Embase, and Scopus. Additional studies were identified through manual screening of the reference lists of included articles and relevant systematic reviews.

The search strategy was structured around three core conceptual domains: model terms, intervention terms, and disease terms. Boolean operators were used to combine these domains, yielding a standardized search string of the form:

(“mathematical model*”) AND (“soil-transmitted helminth*”) AND (“control strateg*”)

The asterisk (*) was used as a truncation operator to capture word variants. Synonyms and related terms were incorporated to increase search sensitivity. The complete database-specific search strategies are provided in the Supporting Information (S2 File).

### Eligibility criteria

The search was restricted to studies published between 2015 and 2024 to capture recent advances in mathematical modelling relevant to STH morbidity control and transmission interruption. This ten-year window ensures alignment with current global health priorities, particularly the WHO roadmaps for 2011–2020 and 2021–2030, while maintaining a manageable scope.

The inclusion criteria were:

a Mathematical models of STH transmission dynamics, including differential equation models (ordinary, partial, or delay), difference equation models, individual-based models, stochastic process models, and hybrid approaches.
b Models evaluating one or more STH control strategies.
c Studies reporting STH model-based outcomes such as infection prevalence, intensity, or elimination thresholds.
d Peer-reviewed journal articles published in English between January 2015 and December 2024.

The exclusion criteria were:

a Non-dynamic STH models (example purely statistical analyses, regression models, or machine learning approaches without transmission dynamics).
b Studies focusing exclusively on cost-effectiveness without incorporating transmission dynamics.
c Diagnostic-focused models addressing only test performance without evaluating transmission or interventions.
d Models of helminth species other than STH.
e Any study that does not evaluate STH control interventions.
f Grey literature (such as dissertations, reports, conference proceedings, abstracts, letters, books, and book chapters).
g Within-host or pharmacological models lacking population-level transmission dynamics.

### Study selection and consistency check

All records retrieved from the databases were imported into EndNote by a single investigator (LM) for duplicate removal and reference management, and subsequently transferred to the Systematic Review Accelerator for additional duplicate screening. The database search identified 597 records, with an additional 14 records obtained through reference list screening, yielding a total of 611 records (see S3 File). After removal of 84 duplicates, 527 unique articles remained for screening.

**Fig 1:**
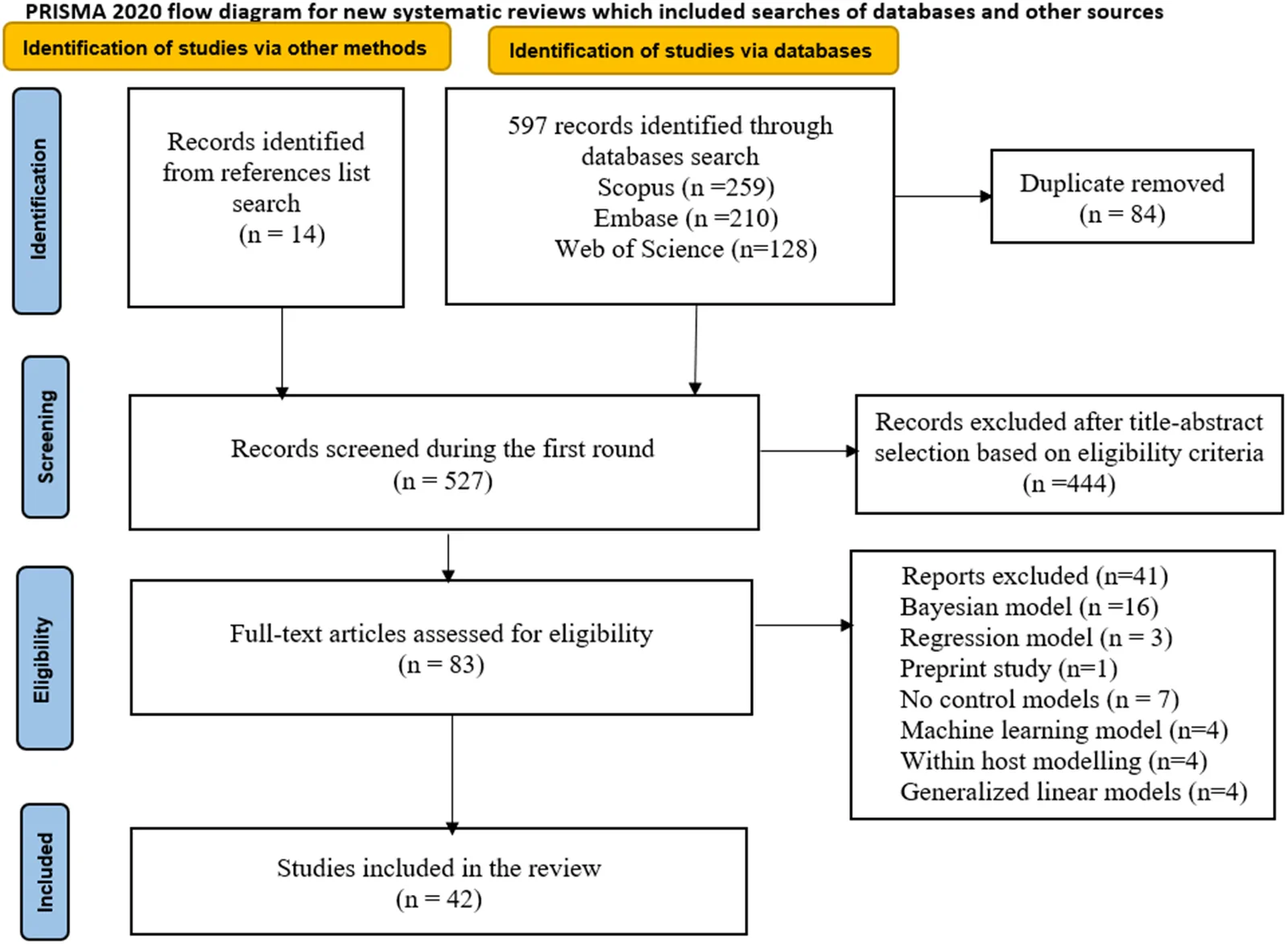
PRISMA schematic diagram showing study selection process. Database searches yielded 597 records; reference searches added 14 records. After removing 84 duplicates, 527 records were screened, 444 excluded, 83 full-text articles assessed, 41 excluded, and 42 studies finally included.

Screening was conducted in two stages by five reviewers (LM, MA, SM, IM, SK). In the first stage, three reviewers (LM, MA, SM) independently screened titles and abstracts of all 527 records against the predefined eligibility criteria. Of these, 444 were excluded, and 83 articles were retained for full-text review. In the second stage, two reviewers (IM, SK) independently assessed the full texts, excluding 41 studies and yielding 42 studies for inclusion. Discrepancies at any stage were resolved through discussion, with a third reviewer consulted where necessary. The study selection process is illustrated in the PRISMA flow diagram (Fig 1).

### Data extraction process, coding of framework, and data synthesis

Data extraction was conducted using Microsoft Excel. The 42 included studies were distributed among five authors: two authors extracted data from nine studies each, and three authors extracted data from eight studies each (LM: 9, MA: 9, SM: 8, IM: 8, SK: 8). Extraction was performed independently between March 2 and March 25, 2025. Furthermore, a reviewer (LM) independently verified a random 10% sample of the extracted data to ensure accuracy. The complete extraction file, including all variables and extractor information, is provided in S4 File.

To ensure consistent classification across heterogeneous studies, we developed and applied a structured coding framework covering model characteristics, intervention characteristics, and the operational incorporation of WHO roadmap targets and reported timelines. Classification categories were defined a priori for the principal model and intervention dimensions, with mutually exclusive study-level categories applied where specified. Methodological characteristics and forms of WHO target incorporation were recorded separately and could be non-mutually exclusive where a study reported more than one approach or analytical role.

#### Model characteristics.

We developed a standardized classification framework based on three dimensions: transmission unit, mathematical framework, and population structure. The transmission unit was used to distinguish between host-based and parasite-based models. Models were classified as host-based if they represented infection dynamics at the level of individual hosts and incorporated depletion of the susceptible population as a negative feedback mechanism. Models were classified as parasite-based if the fundamental unit of transmission was an individual parasite or mated pair, with dynamics represented through the mean of a worm burden distribution.

The mathematical framework was classified as deterministic or stochastic according to the formulation reported in each study. Deterministic models were those using deterministic formulations, whereas stochastic models were those incorporating stochastic or random processes. For studies that compared multiple modelling approaches, the model designated by the authors as the primary analytical framework was used for study-level classification.

Population structure was classified as age-structured when the model explicitly incorporated age, either through discrete age categories or a continuous-age formulation, and as homogeneous when no demographic stratification was incorporated and individuals were represented as epidemiologically identical.

#### Intervention characteristics.

Interventions were decomposed into four orthogonal components: intervention strategy, delivery platform, target-population representation, and treatment regimen. Each component captured a distinct feature of how the intervention was specified in the included modelling studies.

Intervention strategy was defined as the primary control approach evaluated in each study. Studies were classified as preventive chemotherapy (PC) when treatment was the sole intervention and as integrated when preventive chemotherapy was combined with one or more complementary interventions, including WASH, hygiene promotion, or health education.

Delivery platform was defined as the mechanism through which preventive chemotherapy was delivered. Studies were classified as school-based delivery (SBD) when treatment was delivered through schools, community-based delivery (CBD) when treatment was delivered through community-level programmes or outreach activities, and mixed delivery (SBD + CBD) when both school- and community-based delivery mechanisms were incorporated.

Target-population representation described how recipients of the primary intervention were represented in each study. Where intervention recipients were explicitly defined by demographic group, studies were classified according to the groups receiving the intervention: WRA, SAC, PSAC+SAC, SAC+Adults, or PSAC+SAC+Adults. WRA represented interventions targeting only women of reproductive age; SAC represented interventions targeting only school-aged children; PSAC+SAC represented interventions targeting both pre-school-aged and school-aged children; SAC+Adults represented interventions targeting school-aged children and adults; and PSAC+SAC+Adults represented interventions in which pre-school-aged children, school-aged children, and adults were explicitly identified as intervention recipients. Studies in which the intervention was applied across the modelled population without separately identifying demographic groups as intervention recipients were classified as community-wide. Thus, PSAC+SAC+Adults and community-wide were distinguished by the representation of intervention recipients rather than simply by the breadth of population coverage: studies explicitly identifying PSAC, SAC, and adults as intervention recipients were classified as PSAC+SAC+Adults, whereas studies applying the intervention across the modelled population without separately specifying these demographic groups were classified as community-wide.

Treatment regimen described the treatment characteristics represented in each study and included drug type (albendazole, mebendazole, or combination) and reported coverage levels (quantitative or qualitative). When studies evaluated multiple intervention scenarios, information on all scenarios was extracted descriptively; however, study-level classifications used in the quantitative summaries were based on the primary intervention scenario identified by the authors.

#### WHO target alignment and reported timelines.

In this systematic review, WHO target alignment was assessed as an indicator of policy relevance rather than model quality, recognising that mathematical models may be developed for a range of scientific, methodological, and operational purposes. The classification assessed whether and how quantitative targets or programme criteria specified in the WHO 2011–2020 and 2021–2030 roadmaps were incorporated into the model specification or analysis.

Studies were classified as explicitly aligned when a WHO-defined quantitative target or programme criterion was operationally incorporated into the model specification or analysis. Two forms of incorporation were distinguished according to the analytical role of the target or criterion: (i) *intervention target or input*, when a WHO quantitative programme criterion specified the intervention level evaluated in the model; and (ii) *epidemiological target or endpoint*, when a WHO quantitative epidemiological threshold was used as the criterion against which modelled epidemiological outcomes were evaluated, including assessment of whether the specified target was attained. Incorporation in either role was sufficient for classification as explicitly aligned. The two forms were recorded separately and were not mutually exclusive, as a study could use a WHO programme criterion to define an intervention scenario and also evaluate the resulting epidemiological outcome against a WHO quantitative target.

For the 2011–2020 roadmap, explicit alignment was assigned when the WHO target of at least 75% preventive chemotherapy coverage among at-risk pre-school-aged children (PSAC) and school-aged children (SAC) was incorporated as the programme coverage target in the intervention specification or analysis. For the 2021–2030 roadmap, explicit alignment was assigned when the WHO elimination-as-a-public-health-problem (EPHP) criterion of less than 2% moderate and heavy-intensity (MHI) infection prevalence among PSAC and SAC, or another quantitative objective specified in the roadmap, was incorporated as an epidemiological endpoint or criterion for assessing target attainment.

Studies were classified as implicitly aligned when the intervention or epidemiological outcome evaluated corresponded to a WHO roadmap objective, but the relevant WHO quantitative target or programme criterion was not incorporated into the model specification or analysis. For the 2011–2020 roadmap, this included studies evaluating morbidity control through preventive chemotherapy without using the WHO 75% coverage target as the programme coverage criterion. For the 2021–2030 roadmap, this included studies evaluating reductions in MHI infection or other outcomes corresponding to EPHP without applying the WHO threshold of less than 2% MHI infection prevalence as the epidemiological endpoint or target-attainment criterion. Studies that did not meet the criteria for either explicit or implicit alignment were classified as not aligned.

Projected timelines were coded separately from WHO target alignment. A projected timeline was recorded when the study authors reported a time interval, number of years, or projected calendar year associated with attainment of a WHO-relevant outcome. Evaluation of an outcome at a pre-specified simulation time was recorded as a projected timeline only when that time was reported as the time to attainment of the relevant outcome. Studies without a reported time to attainment were classified as having no reported projected timeline.

WHO target alignment was classified independently by two reviewers using these coding criteria, with disagreements resolved through consensus. Data extraction was conducted by five reviewers, with a random subset double-extracted to assess consistency.

Due to substantial heterogeneity in model structures, intervention types, outcome measures, and epidemiological assumptions, a formal meta-analysis was not conducted. Instead, a narrative synthesis was used to describe patterns across the coded domains, summarise the distribution of model and intervention characteristics, and describe WHO roadmap alignment and the projected timelines reported by the included studies.

### Assessment of methodological characteristics

As part of the methodological characterization of the included studies, information on model calibration, validation, sensitivity analysis, and uncertainty quantification was extracted as reported by the study authors. For calibration, studies were classified according to the reported parameter-fitting approach, including maximum likelihood estimation, Bayesian inference, nonlinear least squares, direct fitting to empirical or baseline data, and the use of previously calibrated parameter sets. A study was classified as performing calibration or parameter fitting when at least one model parameter was estimated or adjusted by fitting model outputs to empirical or baseline epidemiological data; previously calibrated parameter sets were recorded separately. Calibration approaches were treated as non-mutually exclusive when more than one approach was reported.

External validation was recorded when model predictions were compared with independent data not used for calibration, including data from a different setting, population, or time period. Sensitivity analysis was classified as local, global, or structural, with these categories treated as non-mutually exclusive. Uncertainty quantification was recorded when uncertainty in model outputs was characterised using approaches such as Bayesian posterior intervals or stochastic simulations. Sensitivity analysis and uncertainty quantification were recorded separately, allowing studies to be classified under either or both.

#### Distribution of included studies by publication year.

Publication year was extracted for each included study and summarised descriptively to show the annual distribution of included studies over the review period.

#### Exploratory analyses.

In addition to the pre-specified extraction variables, two exploratory analyses were conducted during evidence synthesis: (i) a descriptive summary of the helminth species represented across the included studies, and (ii) a narrative synthesis of factors reported by study authors in relation to projected timelines. These post-hoc analyses were conducted to provide additional contextual information and were not considered primary review outcomes.

For the species analysis, each study was coded according to the helminth species reported by the authors. Studies considering a single helminth species were coded according to that species, while studies considering multiple helminth species were coded according to the reported species combination. Studies reporting other helminth combinations or co-infections were coded separately according to the combination reported. The multi-species classification recorded only the species represented in each study and did not imply species-specific parameterisation, application of a common model structure to each species in parallel, or explicit modelling of inter-species interactions or co-infection unless these features were reported by the study authors.

### Risk of bias and assessment of study quality

In addition to characterising model structures, interventions, and WHO alignment, the methodological quality of the included studies was assessed using the Assessment of Modelling Studies (AMS) tool [31], which is used to evaluate modelling studies in health and economics.

The adapted AMS tool [32] comprised twelve criteria: aims and objectives (A), intervention comparators (C), outcome measures (O), model structure (assessed whether model structure and supporting schematic representation were adequately reported) (S), parameter specification (P), assumptions (As), Data quality, sensitivity, and/or uncertainty assessment (D), model validation (V), results presentation (R), discussion and interpretation (Di), Reporting of funding sources and/or competing interests (CI), and modelling methodology (M). Domain‑specific scores for each criterion are provided in Table 5.

Each criterion was scored on a three-point scale (0 = absent, 1 = partially present, 2 = fully present) [33]. Total scores (maximum 24) were converted to percentages and categorized as low (≤50%), medium (50<x≤65%), high (65<x≤80%), or very high (> 80%).

### Bibliometric and network analysis

A bibliometric analysis was conducted using VOSviewer (version 1.6.20) to characterize the research landscape of STH modelling studies. Data were extracted from titles, abstracts, and author affiliations of included studies. Term co-occurrence analysis was used to identify dominant research themes, while co-authorship analysis examined collaboration networks among researchers [34].

A minimum threshold of five occurrences was applied for term inclusion. VOSviewer’s clustering algorithm grouped related terms and authors based on co-occurrence patterns. Network and density visualizations were generated to illustrate thematic structures and colaboration intensity. These analyses complemented the qualitative synthesis by providing insights into research trends, intervention focus areas, and collaborative patterns.

### Handling of missing data

During data extraction, several variables were incompletely reported, including predictive timelines, code availability, model validation, calibration, and uncertainty analyses (see S4 File). All data were recorded as reported, and no imputation was performed due to heterogeneity in model structures and outputs. Missing information was explicitly coded as “not reported” and did not constitute grounds for exclusion. These gaps were incorporated into the narrative synthesis as part of the overall assessment of the evidence base.

## Results

### Trends in the number of published modelling studies

**Fig 2:**
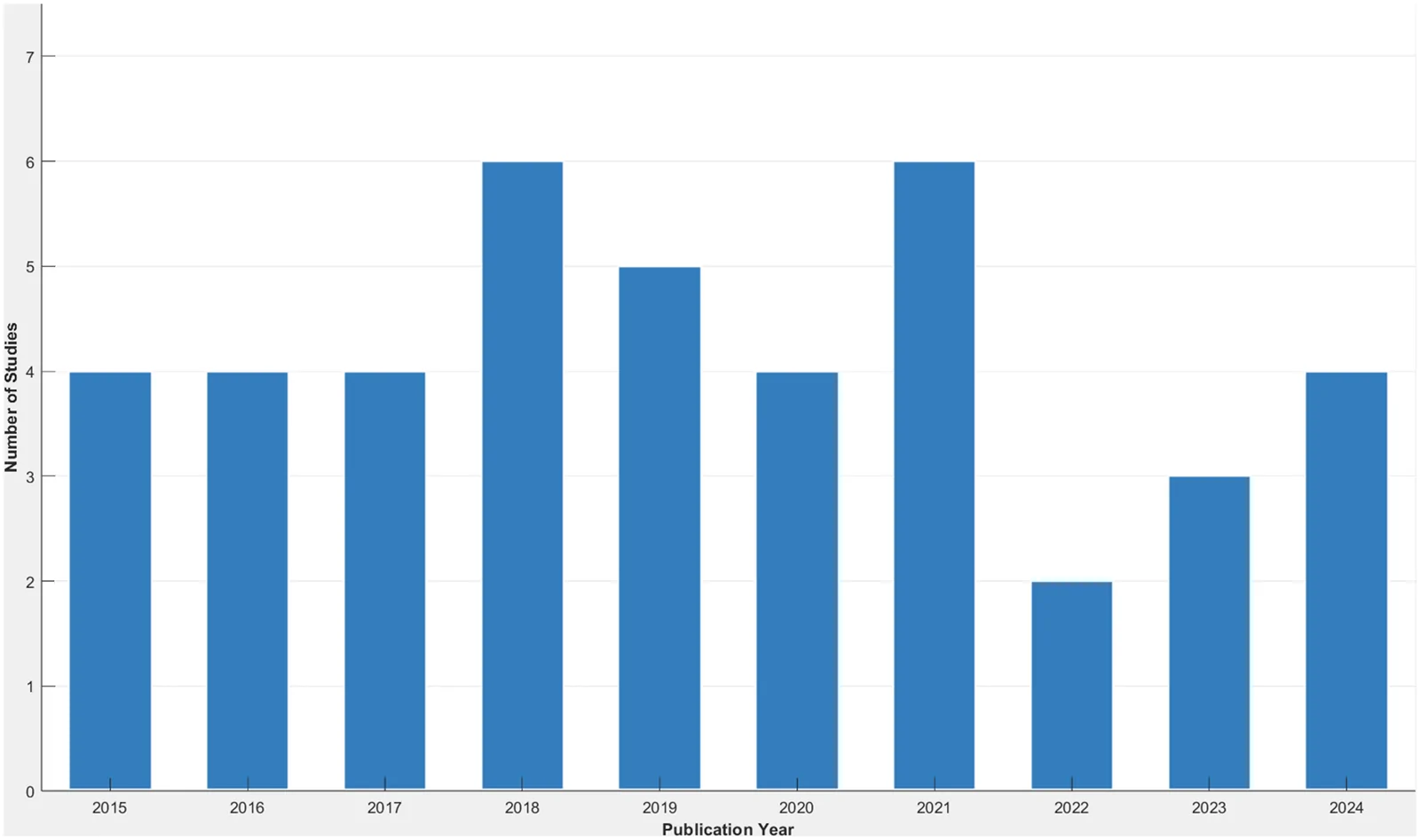
Number of included soil-transmitted helminth (STH) modelling studies by year of publication (2015–2024).

This review analysed 42 mathematical modelling studies published between 2015 and 2024 (Fig 2; Table 1). Four studies (10%) were published in each of 2015, 2016, 2017, 2020, and 2024. Six studies (14%) were published in both 2018 and 2021, while five studies (12%) were published in 2019. Two studies (5%) and three studies (7%) were published in 2022 and 2023, respectively.

**Table 1:** Summary of included studies by author, year, transmission unit, modelling framework, population structure, intervention strategy, and timeline.

Study Characteristics						
Author	Year	TU	F-Work	Population structure	I-strategy	Timeline
Coffeng et al. [35]	2017	Parasite	DM	Age	PC	–
Werkman et al. [36]	2018	Parasite	SD	Age	PC	–
Vegvari et al. [12]	2021	Parasite	SD	Age	PC	–
Mustapha et al. [37]	2020	Host	DM	Flat	Integrated	–
Farrell and Anderson [38]	2018	Parasite	SD	Age	PC	–
Malizia et al. [39]	2020	Parasite	SD	Age	PC	2 yrs
Farrell et al. [40]	2018	Parasite	SD	Age	PC	10 yrs
Vegvari et al. [41]	2021	Parasite	SD	Age	PC	–
Oguntolu et al. [22]	2023	Host	DM	Flat	Integrated	–
Lambura et al. [42]	2020	Host	DM	Flat	PC	–
Lambura et al. [21]	2020	Host	DM	Flat	PC	–
Chazuka et al. [23]	2024	Host	DM	Flat	Integrated	–
Okoyo et al. [43]	2022	Parasite	DM	Age	Integrated	–
Turner et al. [44]	2015	Parasite	DM	Age	PC	2–15 yrs
Clarke et al. [45]	2019	Parasite	SD	Age	PC	–
Hardwick et al. [46]	2019	Parasite	DM	Age	PC	–
Patel et al. [47]	2024	Host	DM	Flat	PC	–
Truscott et al. [48]	2019	Parasite	DM	Flat	PC	–
Chong et al. [49]	2021	Parasite	DM	Flat	PC	3–7 yrs
Kasia and Shengqiang [50]	2016	Host	DM	Flat	PC	–
Anderson et al. [51]	2015	Host	DM	Age	Integrated	–
Vegvari et al. [16]	2019	Parasite	SD	Age	PC	–
Coffeng et al. [52]	2024	Host	DM	Flat	PC	5–6 yrs
Truscott et al. [53]	2017	Parasite	SD	Age	PC	–
Coffeng et al. [13]	2015	Parasite	SD	Age	PC	2 yrs
Truscott et al. [54]	2021	Parasite	SD	Age	PC	2 yrs
Coffeng et al. [55]	2018	Parasite	SD	Age	Integrated	5 yrs
Truscott et al. [56]	2016	Parasite	DM	Age	PC	–
Hardwick et al. [57]	2021	Parasite	SD	Age	PC	–
Collyer and Anderson [58]	2023	Parasite	DM	Flat	PC	–
Chong et al. [59]	2022	Parasite	DM	Flat	PC	–
Maddren et al. [60]	2023	Parasite	SD	Age	PC	2028
Truscott et al. [61]	2015	Parasite	DM	Age	PC	10 yrs
Werkman et al. [62]	2017	Host	DM	Age	PC	–
Okoyo et al. [4]	2021	Parasite	DM	Age	Integrated	5 yrs
Omede et al. [63]	2024	Host	DM	Flat	Integrated	–
Turner et al. [64]	2016	Parasite	DM	Age	PC	2–10 yrs
Werkman et al. [65]	2018	Parasite	SD	Age	PC	–
Davis et al. [66]	2018	Host	DM	Flat	PC	–
Giardina et al. [67]	2019	Parasite	SD	Age	PC	5–6 yrs
Anderson et al. [68]	2017	Parasite	DM	Age	PC	2–3 yrs
Turner et al. [69]	2016	Host	DM	Age	PC	–

**Table notes:** F-work = framework, Transmission unit (TU): Parasite = Parasite‑based model, Host = Host‑based models. Model frameworks: DM = deterministic, SD = stochastic. Population structures: Age = age‑structured, Flat = homogeneous (no stratification). I-strategy = Interventions strategy: PC = preventive chemotherapy, Integrated = preventive chemotherapy combined with one or more complementary interventions (e.g., WASH, health education, or hygiene measures). Timeline indicates a reported time horizon or projected calendar year for achieving a WHO-relevant outcome; “–” indicates that no projected timeline was reported.

### Model taxonomy

**Fig 3:**
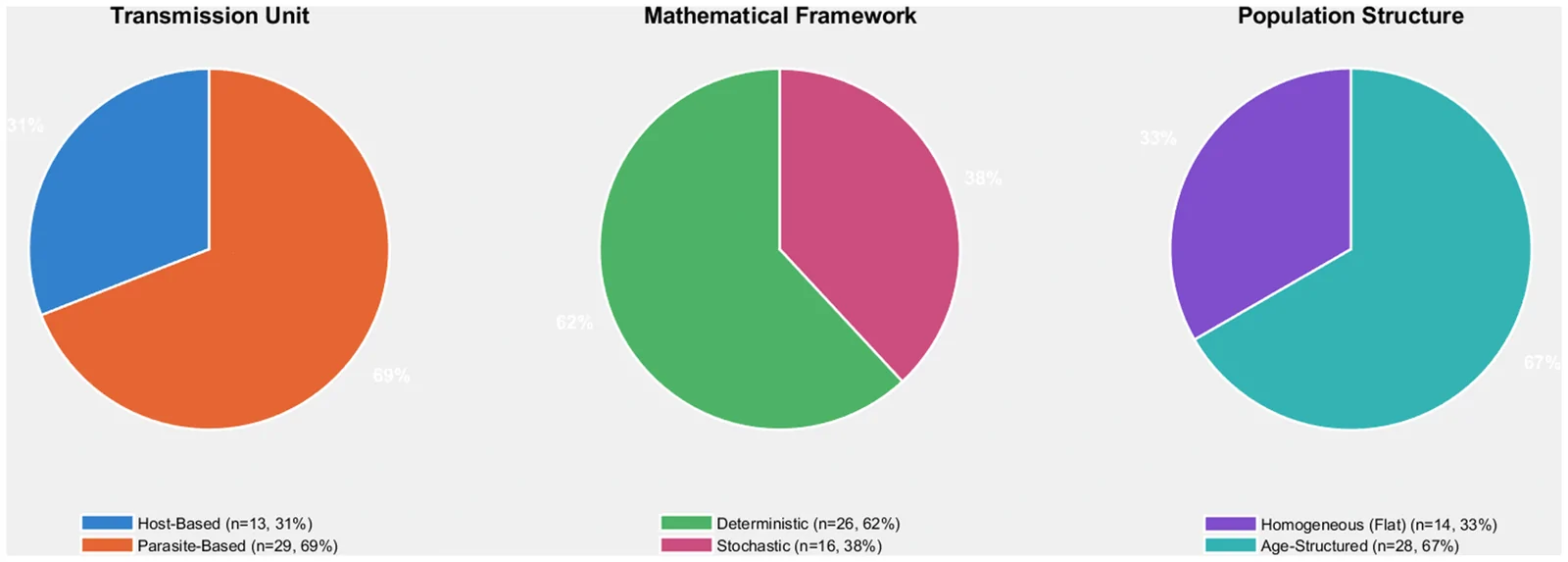
Distribution of model taxonomy characteristics across the 42 included studies, including transmission unit (host- and parasite-based), modelling framework (deterministic and stochastic), and population structure (homogeneous and age-structured).

The distribution of the 42 included studies across the three pre-specified model-taxonomy dimensions is presented below and summarized in Table 2 and Fig 3.

**Table 2:** Summary of model taxonomy across 42 studies. Counts represent number of studies; percentages reflect proportions.

**TRANSMISSION UNIT**		
**Category**	**Count**	**Percentage**
Host-Based	13	31%
Parasite-Based	29	69%
**MATHEMATICAL FRAMEWORK**		
Deterministic	26	62%
Stochastic	16	38%
**POPULATION STRUCTURE**		
Homogeneous (Flat)	14	33%
Age-Structured	28	67%

### Transmission unit

Across the 42 included studies, 13 studies (31%) were classified as host-based models [21–23,37,42,47,50–52,62,63,66,69]. The remaining 29 studies (69%) were classified as parasite-based models [4,12,13,16,35,36,38–41,43–46,48,49,53–61,64,65,67,68].

### Mathematical framework

Across the 42 included studies, 26 (62%) were classified as deterministic models [4,21–23,35,37,42–44,46–52,56,58,59,61–64,66,68,69], while 16 (38%) were classified as stochastic models [12,13,16,36,38–41,45,53–55,57,60,65,67].

### Population structure

Across the 42 included studies, 14 (33%) were classified as homogeneous (flat) models [21–23,37,42,47–50,52,58,59,63,66], while 28 (67%) were classified as age-structured models [4,12,13,16,35,36,38–41,43–46,51,53–57,60–62,64,65,67–69]. Age structure was represented through either discrete age categories (e.g., pre-school-aged children, school-aged children, and adults) or continuous-age formulations using partial differential or integral equation frameworks.

Pie charts (Fig 3) illustrate the distribution of models across the three taxonomic dimensions, including transmission unit, mathematical framework, and population structure

### Intervention decomposition and classification

The distribution of the 42 included studies across the four pre-specified intervention dimensions is presented below and summarised in Table 3.

**Table 3:** Distribution of intervention dimensions across 42 modelling studies.

Dimension	Category	Count	Percentage
Strategy	PC	34	81%
	Integrated	8	19%
Platform	CBD	16	38%
	SBD	6	14%
	SBD + CBD	20	48%
Target-population representation	Community-wide	14	33%
	PSAC + SAC + Adults	17	40%
	PSAC + SAC	4	10%
	SAC	2	5%
	SAC + Adults	2	5%
	WRA	3	7%
Regimen	Single therapy	40	95%
	Dual therapy	2	5%

**Table notes:** PC = preventive chemotherapy; CBD = community-based delivery; SBD = school-based delivery; SBD + CBD = combined school- and community-based delivery; SAC = school-aged children; PSAC = pre-school-aged children; WRA = women of reproductive age. PSAC + SAC + Adults indicates explicit specification of these demographic groups as intervention recipients; Community-wide indicates application of the intervention to a homogeneous modelled population without explicit demographic stratification of recipients. Integrated = PC combined with one or more complementary interventions.

For intervention strategy, 34 studies (81%) were classified as evaluating preventive chemotherapy (PC), while 8 studies (19%) were classified as evaluating integrated strategies combining PC with one or more complementary interventions, such as WASH, health education, hygiene promotion, or shoe wearing.

For delivery platform, 20 studies (48%) were classified as using combined school- and community-based delivery (SBD + CBD), 16 studies (38%) as using community-based delivery (CBD), and 6 studies (14%) as using school-based delivery (SBD).

For target-population representation, 17 studies (40%) explicitly specified PSAC, SAC, and adults as intervention recipients; 14 studies (33%) applied the intervention to a homogeneous modelled population without explicit demographic stratification of intervention recipients; 4 studies (10%) specified both PSAC and SAC; 3 studies (7%) specified WRA; 2 studies (5%) specified SAC only; and 2 studies (5%) specified SAC and adults. Overall, SAC were explicitly represented among the intervention recipients in 25 studies (60%) across the SAC-containing target-population categories.

For treatment regimen, 40 studies (95%) were classified as evaluating single-drug therapy, while 2 studies (5%) were classified as evaluating dual-drug therapy.

### Alignment with WHO targets and reported timelines

Of the 42 included studies, 33 (79%) were classified as aligned with at least one WHO roadmap objective, while 9 (21%) were classified as not aligned. Twelve studies (29%) were classified as explicitly aligned and 21 (50%) as implicitly aligned. Among the explicitly aligned studies, 8 were aligned with the 2011–2020 roadmap and 4 with the 2021–2030 roadmap. Among the implicitly aligned studies, 13 were aligned with the 2011–2020 roadmap and 8 with the 2021–2030 roadmap (Supporting information S4 File).

Among the 12 explicitly aligned studies, 10 operationalised a WHO quantitative programme criterion as an *intervention target or input*, whereby the criterion specified the intervention level evaluated in the model, while 5 operationalised a WHO quantitative epidemiological threshold as an *epidemiological target or endpoint* against which modelled epidemiological outcomes were evaluated. These forms of incorporation were not mutually exclusive, as a study could use a WHO programme criterion to define an intervention scenario and also evaluate the resulting epidemiological outcome against a WHO quantitative target.

Among the 21 implicitly aligned studies, 13 evaluated interventions corresponding to the 2011–2020 roadmap objectives without incorporating the WHO quantitative coverage target into the intervention specification or analysis, while 8 evaluated epidemiological outcomes corresponding to the 2021–2030 roadmap objectives without applying the relevant WHO quantitative endpoint or target-attainment criterion.

**Fig 4:**
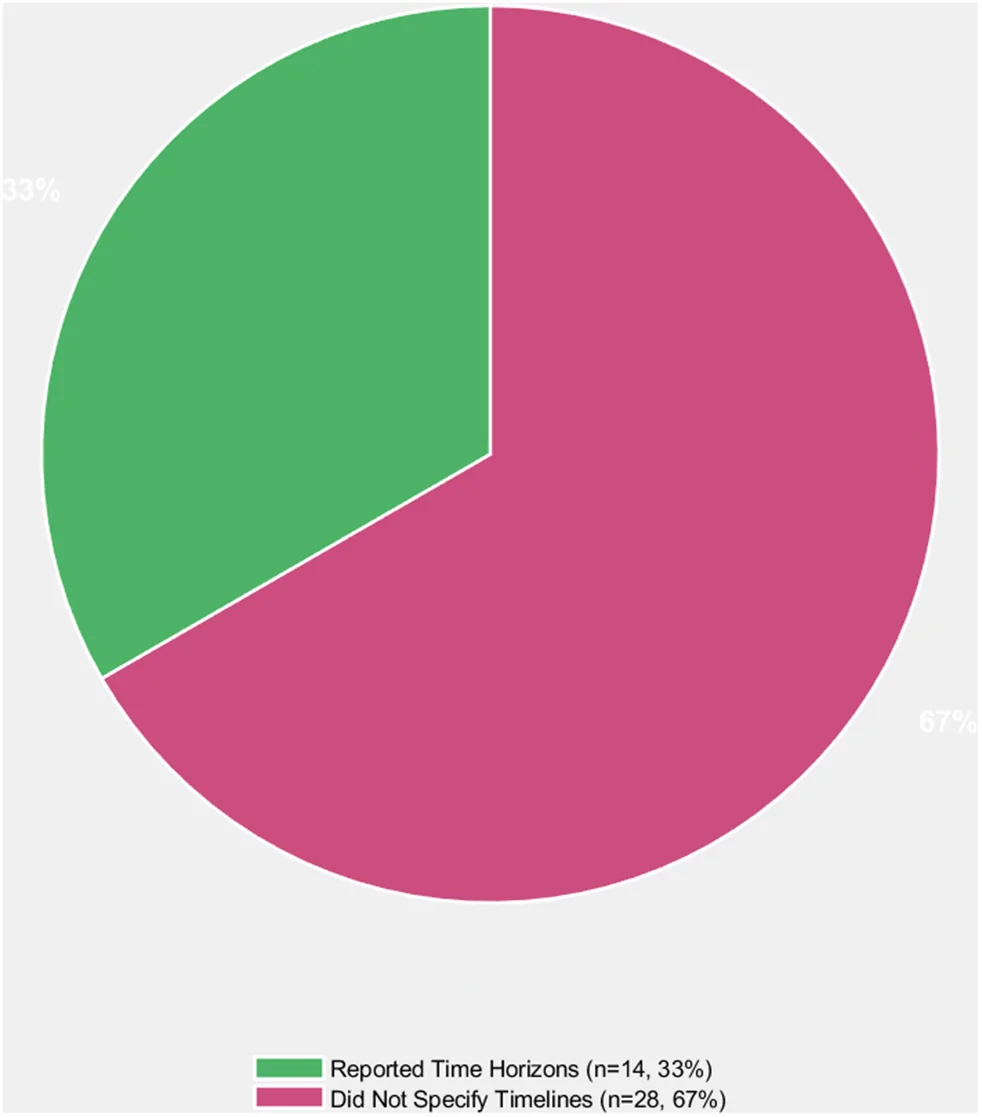
Time-bound projections reported by the included modelling studies. Fourteen studies (33%) reported a time horizon or projected calendar year for a WHO-relevant outcome, whereas 28 studies (67%) did not report a projected timeline.

Among the 42 included studies, 14 (33%) reported a projected timeline for attainment of a WHO-relevant outcome, whereas 28 (67%) did not report a projected timeline (Fig 4; Table 1). Of the 14 studies reporting projected timelines, 7 reported discrete time-to-attainment values (e.g., 2, 5, or 10 years), 6 reported time-to-attainment ranges (e.g., 2–10, 5–6, 3–7, 2–15, and 2–3 years), and 1 reported a projected calendar year (2028) without specifying a relative duration (Table 1).

Among studies aligned with the 2011–2020 roadmap, 9 reported projected timelines, comprising 5 discrete time-to-attainment values and 4 time-to-attainment ranges. Among studies aligned with the 2021–2030 roadmap, 5 reported projected timelines, comprising 2 discrete time-to-attainment values, 2 time-to-attainment ranges, and 1 projected calendar year (2028).

### Factors reported in relation to projected timelines

As part of the post-hoc exploratory analysis, factors reported by study authors in relation to projected timelines were summarised. Among the 14 studies reporting timelines, treatment intensity, including treatment frequency, number of treatment rounds, treatment coverage, or community-wide delivery, was discussed in 11 studies. Drug efficacy was discussed in 5 studies, compliance patterns in 3 studies, and integrated intervention approaches involving preventive chemotherapy combined with WASH-related measures in 3 studies.

Among the remaining 28 studies that did not report projected timelines, reported outcomes included methodological analyses, theoretical investigations, probabilities of elimination, outcomes evaluated at fixed time points, and post-intervention dynamics such as reinfection or rebound patterns.

### Bibliometric analysis

A bibliometric analysis was conducted to examine research themes, co-authorship network, and keyword patterns within the included studies [34].

**Fig 5:**
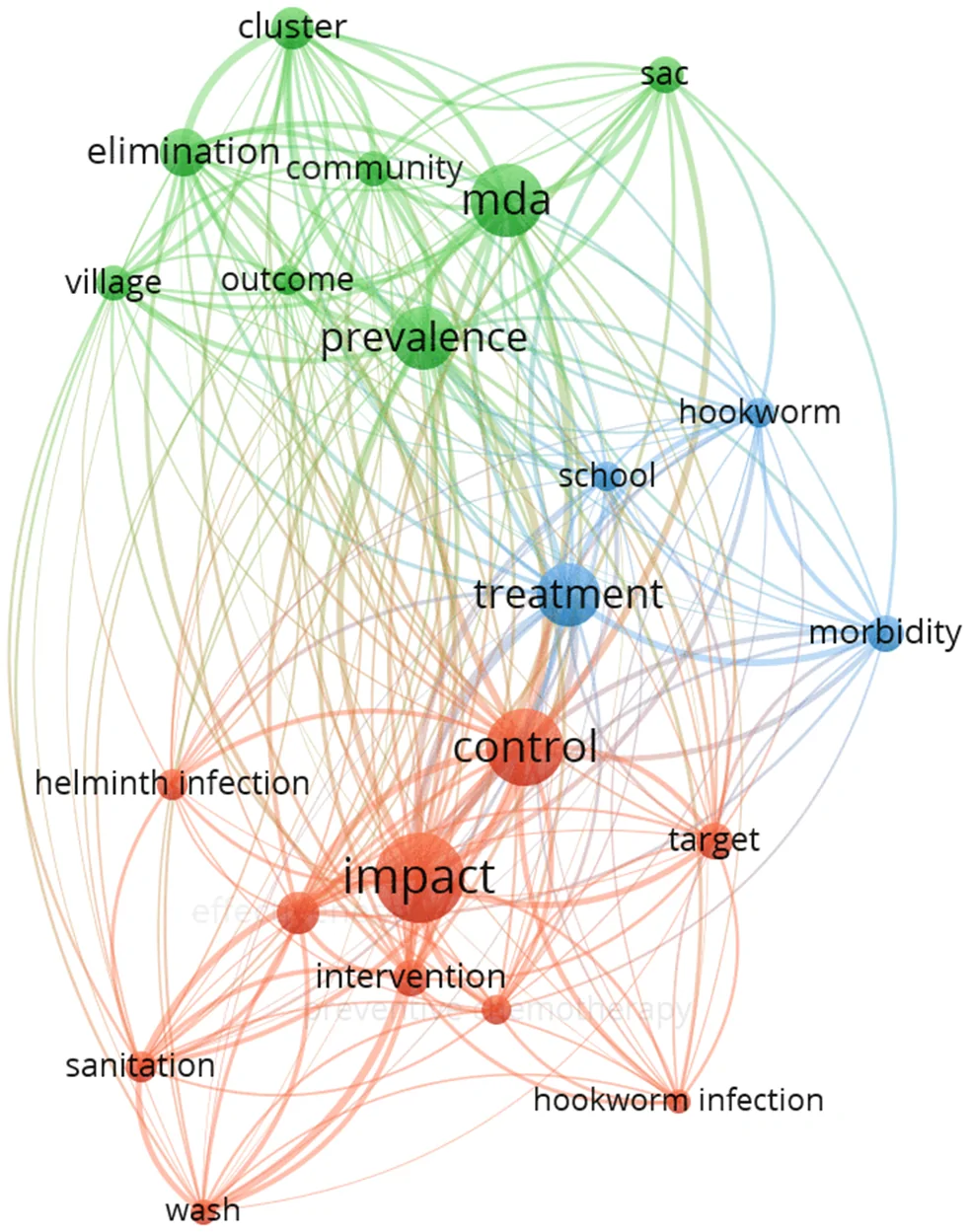
Network visualization of frequently co-occurring terms. Red cluster: helminth infections and targeted interventions; Green cluster: children as the primary target population; Blue cluster: study settings (SAC, clusters, communities, villages).

Fig 5 presents a network visualization of frequently co-occurring terms. Three clusters were identified. One cluster included the terms infection, control, treatment, MDA, and preventive chemotherapy. A second cluster included child, school, SAC, and morbidity. A third cluster included community, village, cluster, and elimination. The terms WASH and sanitation were located outside the three principal clusters.

**Fig 6:**
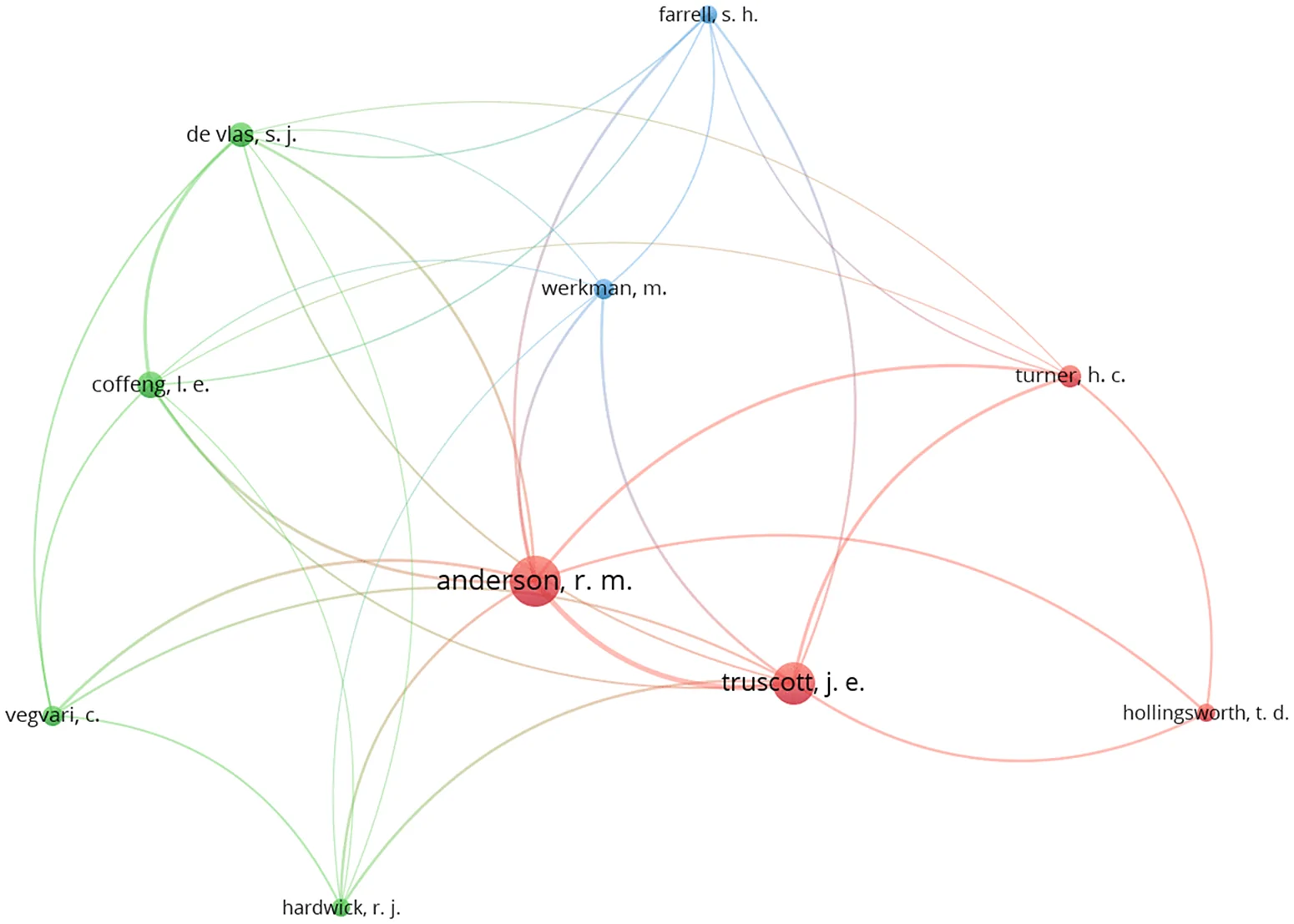
Co-authorship network derived from the included STH modelling studies. Nodes represent authors, with larger nodes indicating higher publication frequency. Links represent co-authorship relationships, with thicker links indicating stronger collaboration. Colors denote clusters of closely collaborating authors identified by the network analysis.

The co-authorship network (Fig 6) identified three collaboration clusters. The largest cluster comprised Anderson, Truscott, Turner, and Hollingsworth. A second independent cluster included Coffeng, de Vlas, Vegvari, and Hardwick. A smaller cluster connected Werkman and Farrell.

**Fig 7:**
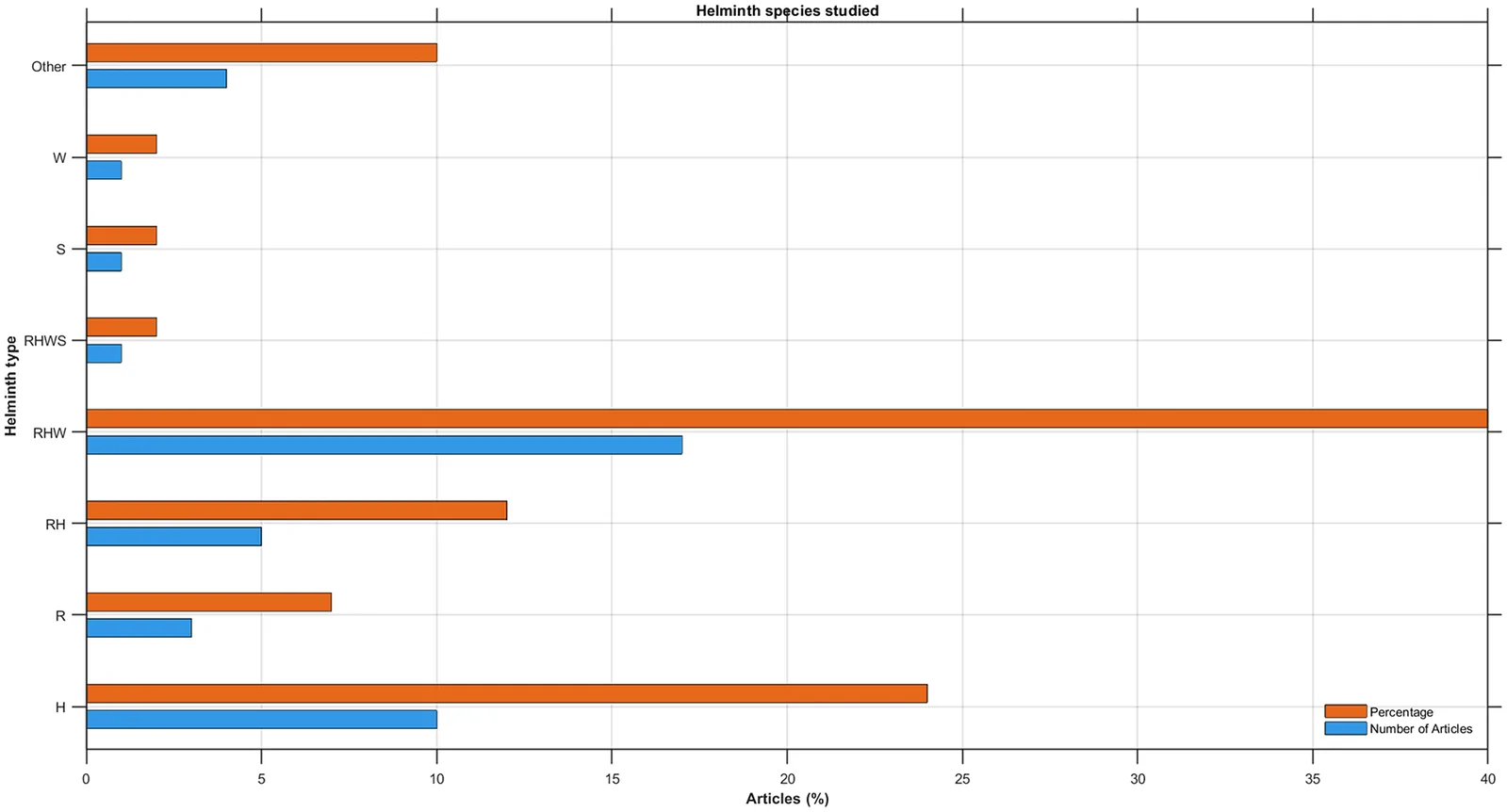
Distribution of helminth species studied across the included modelling articles. H = hookworm, R = roundworm (*Ascaris lumbricoides*), W = whipworm (*Trichuris trichiura*), and S = (*Strongyloides stercoralis*).

As part of the exploratory analysis, Fig 7 summarizes the helminth species represented in the included modelling studies. Of the 42 studies, 10 articles (24%) modelled hookworm only, 3 studies (7%) modelled *Ascaris lumbricoides* only, 1 study (2%) modelled *Strongyloides stercoralis* only, and 1 study (2%) modelled *Trichuris trichiura* only. Mixed-species models were also identified: 5 studies (12%) included hookworm and *Ascaris lumbricoides*, 17 studies (41%) included hookworm, *Ascaris lumbricoides*, and *Trichuris trichiura*, and 1 study (2%) included hookworm, *Ascaris lumbricoides*, *Trichuris trichiura*, and *Strongyloides stercoralis*. Four studies (10%) considered other helminth or co-infection combinations, including *Ancylostoma duodenale* with *Ostertagia*, *Ascaris lumbricoides* with *Schistosoma mansoni*, hookworm with lymphatic filariasis, and STH–TB coinfection. Overall, 23 studies (55%) represented more than one helminth species.

### Methodological characteristics

Across the 42 included studies, methodological characteristics relating to model calibration, validation, sensitivity analysis, and uncertainty quantification were examined. The distribution of these methodological approaches is summarised in Table 4.

**Table 4:** Summary of methodological approaches across 42 STH modelling studies.

Component	N	%	Common methods reported
Calibration	21	50%	MLE and Bayesian inference
Validation (external)	6	14%	Comparison with independent datasets
Sensitivity analysis	22	52%	Local, global, and structural sensitivity analyses
Uncertainty quantification	7	17%	Bayesian posterior intervals and stochastic simulations

#### Model calibration.

Across the 42 included studies, 21 (50%) reported model calibration or parameter fitting, defined as the estimation or adjustment of at least one model parameter by fitting model outputs to empirical or baseline epidemiological data. The remaining studies either relied on literature-based parameter values or did not report a parameter-fitting procedure.

Among the 21 studies reporting calibration or parameter fitting, maximum likelihood estimation (MLE) was reported in 8 studies [4,35,36,43,56,61,62,67]. Bayesian inference was reported in 8 studies, using approaches including Markov chain Monte Carlo (MCMC), approximate Bayesian computation (ABC), and sequential Monte Carlo (SMC/ABC-SMC) [35,48,53,54,56,58,66,68]. Nonlinear least squares (NLS) was reported in 2 studies [37,50], while 3 studies reported direct fitting to empirical or baseline data without specifying a formal statistical framework. Two studies used previously calibrated parameter sets from earlier work [39,40]. The calibration approaches were non-mutually exclusive.

#### Model validation.

Across the 42 included studies, 6 (14%) conducted external validation [4,13,50,52,61,66]. External validation involved comparison of model predictions with independent data not used for calibration, including data from a different setting, population, or time period. A further 16 studies (38%) did not perform independent validation within the study but referenced prior validation of the same or closely related models. The remaining 20 studies (48%) did not report validation or did not specify a validation procedure.

#### Sensitivity analysis and uncertainty quantification.

Across the 42 included studies, 22 (52%) conducted sensitivity analysis. Of these, 19 reported local sensitivity analysis, 4 reported global sensitivity analysis [13,35,43,68], and 5 reported structural sensitivity analysis [12,16,35,39,41]. The sensitivity-analysis categories were non-mutually exclusive, with three studies reporting more than one approach.

Uncertainty quantification (UQ) was reported in 7 of the 42 included studies (17%), including approaches such as Bayesian posterior intervals and stochastic simulations. Three studies reported both sensitivity analysis and uncertainty quantification.

### Results on quality assessment of included studies

Quality assessment scores ranged from 70.8% to 95.8% across the 42 included studies. Based on the pre-defined quality categories, 26 studies (62%) were classified as very high quality (>80%), while 16 studies (38%) were classified as high quality (65<x≤80%). No studies were classified as medium quality (50<x≤65%) or low quality (≤50%).

The criteria most frequently satisfied across the included studies were clear aims (100%), outcome definition (100%), and modelling methods (100%) (Table 5).

**Table 5:** Quality assessment of included studies using the modified Assessment for Modelling Studies (AMS) tool.

Quality Assessment of Included Studies															
Author	A	C	O	S	CI	P	As	D	V	R	Di	M	Total	%	Quality
Clarke et al. [45]	2	2	2	0	2	2	2	2	0	2	2	2	20	83.3	Very high
Coffeng et al. [35]	2	2	2	0	2	1	2	1	2	2	2	2	20	83.3	Very high
Werkman et al. [65]	2	2	2	1	2	2	2	1	0	2	2	2	20	83.3	Very high
Vegvari et al. [12]	2	2	2	0	2	2	2	2	0	2	2	2	20	83.3	Very high
Mustapha et al. [37]	2	2	2	2	2	2	1	2	0	2	2	2	21	87.5	Very high
Farrell et al. [38]	2	1	2	1	2	1	2	2	0	1	2	2	18	75.0	High
Malizia et al. [39]	2	2	2	1	2	2	2	2	0	2	2	2	21	87.5	Very high
Farrell et al. [40]	2	2	2	1	2	2	2	2	0	2	2	2	21	87.5	Very high
Vegvari et al. [41]	2	2	2	1	2	1	2	1	0	2	2	2	19	79.2	High
Oguntolu et al. [22]	2	1	2	2	2	2	1	1	0	2	2	2	19	79.2	High
Lambura et al. [42]	2	2	2	2	2	1	2	1	0	2	2	2	20	83.3	Very high
Lambura et al. [21]	2	2	2	2	2	2	2	1	0	2	2	2	21	87.5	Very high
Chazuka et al. [23]	2	2	2	2	2	2	2	1	0	2	2	2	21	87.5	Very high
Okoyo et al. [43]	2	2	2	1	2	2	2	2	0	2	2	2	21	87.5	Very high
Turner et al. [44]	2	2	2	1	2	1	2	2	0	2	2	2	20	83.3	Very high
Hardwick et al. [46]	2	2	2	1	2	2	1	1	0	2	2	2	19	79.2	High
Patel et al. [47]	2	1	2	1	2	2	1	0	0	2	2	2	17	70.8	High
Truscott et al. [48]	2	2	2	1	2	2	1	2	0	2	2	2	20	83.3	Very high
Chong et al. [49]	2	1	2	1	2	1	2	1	0	2	2	2	18	75.0	High
Kasia et al. [50]	2	2	2	2	2	1	2	2	0	2	2	2	21	87.5	Very high
Vegvari et al. [16]	2	2	2	1	2	1	2	1	0	2	2	2	19	79.2	High
Coffeng et al. [52]	2	2	2	1	2	2	1	2	0	2	2	2	20	83.3	Very high
Truscott et al. [53]	2	2	2	1	2	1	2	2	0	2	2	2	20	83.3	Very high
Coffeng et al. [13]	2	2	2	1	2	2	1	2	2	2	2	2	22	91.7	Very high
Truscott et al. [54]	2	1	2	1	2	2	2	2	0	2	2	2	20	83.3	Very high
Turner et al. [64]	2	1	2	1	2	2	2	2	0	2	2	2	20	83.3	Very high
Coffeng et al. [55]	2	2	2	1	2	1	2	2	0	2	2	2	20	83.3	Very high
Truscott et al. [56]	2	1	2	1	2	2	2	2	2	1	2	2	21	87.5	Very high
Hardwick et al. [57]	2	1	2	1	2	2	2	1	0	2	2	2	19	79.2	High
Collyer et al. [58]	2	1	2	2	2	1	1	2	0	2	2	2	19	79.2	High
Chong et al. [59]	2	2	2	1	2	2	1	2	0	2	2	2	20	83.3	Very high
Maddren et al. [60]	2	2	2	0	2	2	1	2	0	1	2	2	18	75.0	High
Truscott et al. [61]	2	2	2	1	2	1	2	2	2	2	2	2	22	91.7	Very high
Werkman et al. [62]	2	2	2	1	2	2	1	1	0	2	2	2	19	79.2	High
Okoyo et al. [4]	2	2	2	2	2	1	2	2	2	2	2	2	23	95.8	Very high
Omede et al. [63]	2	1	2	2	2	1	0	1	0	2	2	2	17	70.8	High
Turner et al. [69]	2	2	2	1	2	1	2	1	0	2	2	2	19	79.2	High
Werkman et al. [36]	2	2	2	1	2	1	1	1	0	2	2	2	18	75.0	High
Davis et al. [66]	2	2	2	2	2	2	2	1	2	2	2	2	23	95.8	Very high
Giardina et al. [67]	2	2	2	1	2	2	1	1	0	2	2	2	19	79.2	High
Anderson et al. [68]	2	2	2	0	2	2	1	2	0	2	2	2	19	79.2	High
Anderson et al. [51]	2	2	2	1	2	2	2	1	0	2	2	2	20	83.3	Very high

**Table notes:** AMS criteria abbreviations: A = Clear aims and objectives; C = Intervention comparators; O = Outcome measure defined; S = Model structure & flow chart; CI = Reporting of funding sources and/or competing interests; P = Parameters specified; As = Assumptions justification; D = Data quality, sensitivity, and/or uncertainty; V = Model validation; R = Results presentation; Di = Results discussion & interpretation; M = Modelling methods.

## Discussion

This systematic review synthesised 42 mathematical modelling studies of soil-transmitted helminth (STH) control published between 2015 and 2024, focusing on model structure, intervention representation, methodological characteristics, operational incorporation of WHO roadmap targets, and reported projected timelines. Taken together, the findings characterise a modelling literature in which parasite-based, age-structured, and deterministic formulations were frequently represented and preventive chemotherapy (PC) formed the principal intervention focus. Although many studies addressed WHO-relevant interventions or outcomes, explicit operational incorporation of WHO quantitative targets or programme criteria and reporting of projected timelines were less frequent. External validation and uncertainty quantification were also less frequently reported. These patterns provide a basis for considering not only how STH transmission and control have been modelled, but also how the resulting models have been applied to programme-relevant questions.

The model taxonomy showed that parasite-based, age-structured, and deterministic formulations were the most frequently represented approaches in the reviewed literature. Taken together, this pattern indicates frequent representation of parasite-level processes and demographic heterogeneity within models that primarily describe expected population-level transmission trajectories. The prominence of parasite-based and age-structured formulations is consistent with the importance of worm burden, mating probability, density-dependent fecundity, and age-dependent variation in exposure and infection intensity in STH transmission [4,13,61]. These characteristics are particularly relevant to modelling infection intensity, age-targeted preventive chemotherapy, and transmission responses across population groups. Deterministic formulations provide a complementary population-level perspective by describing expected transmission and intervention trajectories under specified assumptions, whereas stochastic approaches can additionally characterise variability in trajectories and probabilities of outcomes such as elimination or resurgence [70–72]. The comparatively lower representation of host-based, homogeneous, and stochastic formulations therefore indicates that these alternative levels of biological, demographic, and probabilistic representation were less frequently used. This distribution should not be interpreted as evidence that one modelling approach is preferable to another; rather, it highlights the importance of matching the transmission unit, population structure, and mathematical framework to the epidemiological or intervention question being addressed.

The relevance of these structural choices becomes particularly apparent in how interventions are represented. Preventive chemotherapy was the predominant strategy in the reviewed models, whereas integrated interventions were comparatively less frequent. Beyond intervention strategy, the models varied in their representation of delivery platforms, target populations, and treatment regimens. Interventions explicitly spanning PSAC, SAC, and adults, as well as those applied to homogeneous community-wide populations, were frequently represented, while delivery incorporating both school- and community-based approaches was more common than either delivery platform alone. Although SAC-only interventions were comparatively less frequent, SAC were commonly represented within broader target-population configurations involving PSAC and/or adults. Thus, the small number of SAC-only classifications reflects how intervention recipients were grouped rather than limited representation of SAC in the reviewed models. Single-drug therapy also accounted for most modelled treatment regimens.

Taken together, these patterns may indicate that the reviewed literature has primarily examined treatment-based STH control across different target populations, delivery approaches, and treatment regimens. This emphasis may be consistent with the central role of PC in STH control programmes and its implementation across different at-risk populations and delivery settings [5,14,15]. In contrast, PC combined with complementary measures such as WASH, hygiene promotion, or health education has been less frequently examined. Given the emphasis on integrated approaches in the WHO 2021–2030 NTD roadmap [14,15], further modelling of such combinations may broaden the range of programme-relevant intervention questions that can be evaluated. As with model structure, however, the appropriate intervention representation depends on the programme question being investigated.

The programme relevance of these intervention analyses also depends on how policy objectives are translated into model inputs and outcomes. Most reviewed studies were related to at least one WHO roadmap objective, but implicit alignment was more frequent than explicit operational incorporation of WHO quantitative targets or programme criteria. Among explicitly aligned studies, WHO targets were used predominantly to specify intervention scenarios or inputs, while fewer studies incorporated them as epidemiological endpoints or criteria for assessing target attainment. This pattern may indicate that WHO-relevant interventions and outcomes have been widely represented, while quantitative programme targets have less frequently been used to evaluate projected outcomes directly against specified thresholds. These forms of incorporation serve different analytical purposes: programme targets used as intervention inputs define the conditions under which an intervention is evaluated, whereas their use as epidemiological endpoints or target-attainment criteria allows model projections to be assessed in relation to a specified programme outcome. Where evaluation of progress towards a defined WHO target is an intended modelling objective, explicit use of the relevant quantitative criterion may therefore provide a clearer basis for interpreting projected outcomes in relation to that target.

A related programme question is not only whether a specified outcome can be reached, but also when it may occur. Projected timelines were reported by only a subset of the reviewed studies. Treatment intensity, drug efficacy, compliance assumptions, and integrated intervention approaches were among the factors reported in relation to these timelines. The variation across studies suggests that projected time to a specified outcome is conditional on the epidemiological, treatment, and behavioural assumptions represented in individual models and should therefore not be interpreted independently of those assumptions. Reporting projected timelines may provide additional information for time-bound control or elimination planning when target attainment and its timing are central research questions. Their comparatively limited reporting does not, however, imply that studies without timeline estimates lack programme relevance, since many models were developed for transmission, intervention, or methodological questions for which time to a programme target was not the intended outcome.

The interpretation of such model projections also depends on how model performance and uncertainty are assessed. Calibration and sensitivity analysis were more frequently reported than external validation and uncertainty quantification. This pattern may suggest that the reviewed literature has placed greater emphasis on fitting model parameters and examining model responses to changes in inputs or assumptions than on evaluating predictions against independent data or formally characterising uncertainty in projected outcomes. These approaches provide different forms of information about model performance and robustness: calibration assesses agreement with data used for model fitting, sensitivity analysis examines how changes in inputs or assumptions affect model outputs, external validation evaluates predictions against independent observations, and uncertainty quantification characterises uncertainty surrounding model estimates or projections [73]. The comparatively limited reporting of external validation and uncertainty quantification may constrain assessment of predictive performance and the uncertainty surrounding model projections. Where prediction or programme evaluation is an intended application, independent validation and appropriate uncertainty quantification could therefore strengthen the interpretation of projected outcomes.

Beyond these principal structural, intervention, and programme-related findings, the exploratory analyses provide additional context on the scope of the literature. The post-hoc species analysis showed that hookworm, *Ascaris lumbricoides*, and *Trichuris trichiura* were the species most frequently represented, whereas other species and species combinations appeared less frequently. This distribution indicates that the modelling literature reviewed here has largely centred on the major STH species commonly addressed by control programmes. Representation of multiple species within a study varied in form and did not necessarily involve explicit modelling of inter-species interactions or co-infection. Given its exploratory nature, this analysis could therefore be interpreted as characterising the taxonomic coverage of the reviewed literature rather than the complexity of species interactions represented within individual models.

The bibliometric findings provide a complementary perspective on these substantive patterns. Terms related to preventive chemotherapy, treatment, infection, and elimination were prominent, consistent with the treatment focus identified through the intervention classification, whereas WASH-related terminology was less prominent. The co-authorship network also showed recurring clusters of collaboration among modelling groups. Thus, the thematic structure of the literature broadly reinforces the intervention patterns identified through the structured review, while the collaboration network provides additional context on how this body of modelling research is organised.

Overall, the findings characterise an STH modelling literature in which parasite-based, age-structured, and deterministic formulations are frequently represented, preventive chemotherapy remains the principal intervention focus, and WHO-relevant interventions and outcomes are more commonly represented than the explicit operational use of quantitative programme targets or estimation of time to target attainment. Methodological assessment further indicates that calibration and sensitivity analysis are more frequently reported than external validation and uncertainty quantification. These patterns could be interpreted in relation to the scientific or programme questions addressed by individual models rather than as indicators of methodological superiority or model quality. Where programme-target evaluation or planning is an intended application, explicit incorporation of relevant programme criteria, appropriate uncertainty assessment, independent validation where suitable data are available, and estimation of time-dependent outcomes could strengthen the interpretation of model projections for their intended use.

### Recommendations

Based on the synthesis of the 42 included studies, several implications emerge for future model development, reporting, and programme-relevant applications. Given the heterogeneity in model structures, intervention designs, and epidemiological assumptions, these recommendations should be interpreted in relation to the intended research or programme question rather than as universal modelling requirements.

For modelling research, the predominance of parasite-based, age-structured, deterministic, and preventive-chemotherapy-focused models highlights the modelling approaches and intervention questions most frequently represented in the reviewed literature. Alternative structures may provide complementary information where required by the research question. In particular, stochastic approaches may be informative when elimination probabilities, resurgence, or transmission at low prevalence are of interest. The comparatively limited representation of integrated intervention strategies also indicates scope for further evaluation of preventive chemotherapy in combination with complementary measures such as WASH, health education, and hygiene-related interventions.

For models intended to inform programme planning or policy evaluation, WHO-defined quantitative targets or programme criteria may be incorporated according to their intended analytical role, including as intervention scenarios or inputs, epidemiological endpoints, or criteria for assessing target attainment. Where time to a specified programme outcome is an intended model output, projected timelines could be reported together with the epidemiological and intervention assumptions underlying those projections. Appropriate uncertainty quantification and external validation, where independent data are available, may further strengthen the interpretation of model projections for their intended applications.

For programme implementation and policy, greater availability of longitudinal programme data following deworming interventions could facilitate independent evaluation of model predictions and assessment of projected programme outcomes. Such data may strengthen the empirical basis for models intended to evaluate STH control and elimination strategies.

### Limitations

This review should be interpreted in light of several methodological considerations. The search was restricted to English-language peer-reviewed articles published between 2015 and 2024 and indexed in Web of Science, Scopus, or Embase. Consequently, relevant studies published before 2015, in other languages, in the grey literature, or indexed exclusively in other databases may have been missed.

Substantial heterogeneity in modelling frameworks, intervention scenarios, outcome measures, and reporting precluded formal meta-analysis; consequently, the findings are based on narrative synthesis. Differences in model structure, assumptions, parameterisation, intervention design, and outcome definitions also limit direct comparison of projections across studies. The review therefore characterises patterns in the modelling literature rather than providing pooled estimates of intervention effectiveness or time to target attainment.

The development and application of the model taxonomy, intervention decomposition framework, and WHO target-alignment classification involved some judgement in assigning studies to predefined categories. Explicit coding rules and reviewer cross-checking were used to promote consistency, but alternative operational definitions may produce different classifications. For WHO alignment specifically, intervention scenario/input and epidemiological endpoint were recorded as distinct analytical roles within explicit alignment and were not interpreted as equivalent uses of a WHO quantitative target or programme criterion.

The review also relied on information reported in the included publications. Incomplete reporting may therefore have resulted in some methodological features being classified as not reported even when they may have been undertaken but not described in the publication. External validation, uncertainty quantification, and projected timelines were reported in only subsets of the included studies; consequently, the review can characterise the extent to which these features were reported but cannot infer their absence where reporting was insufficient or establish the predictive performance or robustness of models for which such assessments were not reported.

## Conclusion

This systematic review synthesised mathematical modelling studies of soil-transmitted helminthiasis published between 2015 and 2024 to characterise model structures, intervention representation, methodological practices, operational incorporation of WHO roadmap targets, and reported projected timelines. The reviewed literature was predominantly characterised by parasite-based, age-structured, and deterministic formulations, with preventive chemotherapy forming the principal intervention focus. Integrated intervention strategies were comparatively less represented, while WHO-relevant interventions and outcomes were more commonly represented than explicit operational incorporation of WHO quantitative targets or programme criteria. External validation, uncertainty quantification, and reporting of projected timelines were also comparatively infrequent.

Overall, the findings provide a structured synthesis of how STH transmission and interventions have been represented in the modelling literature and how WHO targets and programme criteria have been incorporated into model analyses. The observed patterns could be interpreted in relation to the scientific or programme questions addressed by individual models rather than as indicators of methodological superiority or model quality. For models intended to address programme planning, target attainment, or policy-evaluation questions, appropriate representation of integrated interventions where relevant, clearly defined operational use of programme criteria, reporting of projected timelines where estimated, and appropriate validation and uncertainty assessment could strengthen the interpretation of model projections for their intended applications.

## Supporting information

S1 File PRISMA 2020 checklist. (pdf) From: Page MJ, McKenzie JE, Bossuyt PM, Boutron I, Hoffmann TC, Mulrow CD, et al. The PRISMA 2020 statement: an updated guideline for reporting systematic reviews. BMJ 2021;372:n71. https://doi.org/10.1136/bmj.n71.
(PDF)

S2 File Full search strategies or strings. (PDF)

S3 File Data extraction excel sheet for all excluded and included studies. (XLSX)

S4 File Data extraction excel sheet. (XLSX)

S5 File Review Protocol. (PDF)
