# Supplementary material for: A systematic review of mathematical models for soil-transmitted helminthiasis control strategies
Source: PLoS Negl Trop Dis. 2026 Aug 28;20(8):e0013431. doi: 10.1371/journal.pntd.0013431 (PMC13537693; doi:10.1371/journal.pntd.0013431)
Supplement: Supplementary file 1 [file pntd.0013431.s001.pdf]

S1 Table: PRISMA 2020 Checklist – Review full text and abstract

Section and Topic	Item #	Checklist item	Location where item is reported
**TITLE**			
Title	1	Identify the report as a systematic review.	Page 1. A Systematic Review of Mathematical Models for Helminthiasis Control Strategies
**ABSTRACT**			
Abstract	2	See the PRISMA 2020 for Abstracts checklist.	Page 1-2. Abstract includes objectives, methods, results, and conclusions.
**INTRODUCTION**			
Rationale	3	Describe the rationale for the review in the context of existing knowledge.	Page 3. Existing reviews focused broadly on NTD control without systematically synthesizing STH transmission models. This review addresses the gap by (i) systematically classify models based on their biological and structural characteristics; (ii) decompose STH control interventions represented in mathematical models into a set of orthogonal components and use these to develop a systematic classification framework; and (iii) synthesize model predictions in relation to WHO-defined targets and timelines.
Objectives	4	Provide an explicit statement of the objective(s) or question(s) the review addresses.	Page 3. To (i) classify STH transmission models by biological and structural characteristics; (ii) decompose interventions into orthogonal components; and (iii) synthesize model predictions in relation to WHO 2011–2020 and 2021–2030 targets and timelines.
**METHODS**			
Eligibility criteria	5	Specify the inclusion and exclusion criteria for the review and how studies were grouped for the syntheses.	Page 4-5 Inclusion: mathematical models of STH transmission (2015–2024, English, peer-reviewed) evaluating at least one control strategy. Exclusion: non-dynamic models, cost-only studies, grey literature, non-STH helminths. Studies were grouped by model taxonomy (transmission unit, framework, population structure), intervention components (strategy, platform, target, regimen), and WHO target alignment (2011–2020 vs. 2021–2030)
Information	6	Specify all databases, registers, websites, organisations,	Page 4. We searched Web of Science, Scopus, and Embase

S1 Table: PRISMA 2020 Checklist – Review full text and abstract

Section and Topic	Item #	Checklist item	Location where item is reported
sources		reference lists and other sources searched or consulted to identify studies. Specify the date when each source was last searched or consulted.	(From 1 January 2015 to Dec 31, 2024.
Search strategy	7	Present the full search strategies for all databases, registers and websites, including any filters and limits used.	Search query combined terms for mathematical model*+soil-transmitted helminth* +control strateg*. The full search is provided as supplementary.
Selection process	8	Specify the methods used to decide whether a study met the inclusion criteria of the review, including how many reviewers screened each record and each report retrieved, whether they worked independently, and if applicable, details of automation tools used in the process.	Page 5. We composed some pre-defined rule. Five independent reviewers independently screened titles and abstracts for eligibility, resolved conflicts by consensus. Full texts were reviewed for inclusion based on predefined criteria. EndNote and the Systematic Review Accelerator were used to identify and remove duplicates.
Data collection process	9	Specify the methods used to collect data from reports, including how many reviewers collected data from each report, whether they worked independently, any processes for obtaining or confirming data from study investigators, and if applicable, details of automation tools used in the process.	Page 5-8. Five reviewers independently extracted data using a structured Excel sheet. VOSviewer was used for bibliometric analysis. No automation tools were used. A narrative synthesis was conducted due to heterogeneity in models and outcomes.
Data items	10a	List and define all outcomes for which data were sought. Specify whether all results that were compatible with each outcome domain in each study were sought (e.g. for all measures, time points, analyses), and if not, the methods used to decide which results to collect.	Page 8-20. Outcomes sought were: (i) model characteristics (transmission unit, mathematical framework, population structure), (ii) intervention characteristics (strategy, delivery platform, target population, treatment regimen), (iii) alignment with WHO 2011–2020 and 2021–2030 targets, and (iv) reported timelines (years to achieve targets). All results compatible with these domains (e.g., all time points, all intervention scenarios) were extracted without restriction. When multiple scenarios were reported, the primary or policy-relevant scenario was prioritized as defined by the authors.
	10b	List and define all other variables for which data were sought (e.g. participant and intervention characteristics, funding sources). Describe any assumptions made about any missing or	Page 8. Other extracted variables included study characteristics (author, year, publication journal), model features (transmission unit, mathematical framework, population structure),

Section and Topic	Item #	Checklist item	Location where item is reported
		unclear information.	intervention details (strategy, delivery platform, target population, drug regimen), WHO target alignment (2011–2020 or 2021–2030), and reported timelines. The missing information were indicated as not reported and no study excluded for missing such data.
Study risk of bias assessment	11	Specify the methods used to assess risk of bias in the included studies, including details of the tool(s) used, how many reviewers assessed each study and whether they worked independently, and if applicable, details of automation tools used in the process.	Page 8. Risk of bias was assessed using a modified Assessment of Modeling Studies (AMS) tool. four reviewers independently evaluated each study, scoring criteria from 0 to 2 across 12 items, including two added for WHO alignment and predictive timelines. Scores were converted to percentages and categorized into four quality levels.
Effect measures	12	Specify for each outcome the effect measure(s) (e.g. risk ratio, mean difference) used in the synthesis or presentation of results.	Page 7-8. Narrative synthesis; no meta-analysis performed.
Synthesis methods	13a	Describe the processes used to decide which studies were eligible for each synthesis (e.g. tabulating the study intervention characteristics and comparing against the planned groups for each synthesis (item #5)).	Page 7-8. We used table to present all information in regards to the three study objectives. Missing data were coded as “not reported” without imputation; no data conversions were performed due to heterogeneity across model structures and outcomes.
	13b	Describe any methods required to prepare the data for presentation or synthesis, such as handling of missing summary statistics, or data conversions.	Page 8. Data were organized into structured tables categorizing model types, interventions, and outcomes. No data conversions were performed due to heterogeneity; synthesis was narrative.
	13c	Describe any methods used to tabulate or visually display results of individual studies and syntheses.	Page 8-20. Results were tabulated in structured Excel sheets and presented in tables (e.g., Table 1, Table 5) and figures (bar charts, pie charts, network/density visualizations) generated using VOSviewer (Figs 1–9) to display study characteristics, model taxonomy, intervention decomposition, and bibliometric patterns.
	13d	Describe any methods used to synthesize results and provide a rationale for the choice(s). If meta-analysis was performed,	Page 7-8. A narrative synthesis was used due to substantial heterogeneity in model types, interventions, outcomes, and

Section and Topic	Item #	Checklist item	Location where item is reported
		describe the model(s), method(s) to identify the presence and extent of statistical heterogeneity, and software package(s) used.	assumptions across studies, which precluded meta-analysis.
	13e	Describe any methods used to explore possible causes of heterogeneity among study results (e.g. subgroup analysis, meta-regression).	Not applicable
	13f	Describe any sensitivity analyses conducted to assess robustness of the synthesized results.	Not applicable
Reporting bias assessment	14	Describe any methods used to assess risk of bias due to missing results in a synthesis (arising from reporting biases).	Not applicable
Certainty assessment	15	Describe any methods used to assess certainty (or confidence) in the body of evidence for an outcome.	Not applicable
**RESULTS**			
Study selection	16a	Describe the results of the search and selection process, from the number of records identified in the search to the number of studies included in the review, ideally using a flow diagram.	Page 5 and 9. A total of 611 records were identified, 84 duplicates removed, and 527 screened. After screening and full-text review, 42 studies met the inclusion criteria and were included.
	16b	Cite studies that might appear to meet the inclusion criteria, but which were excluded, and explain why they were excluded.	Not applicable
Study characteristics	17	Cite each included study and present its characteristics.	Page 9-10. See table 1
Risk of bias in studies	18	Present assessments of risk of bias for each included study.	Page 18-19. Table 5 presents individual AMS risk of bias scores and quality categories (very high/high) for all 42 included studies.
Results of individual studies	19	For all outcomes, present, for each study: (a) summary statistics for each group (where appropriate) and (b) an effect estimates and its precision (e.g. confidence/credible interval), ideally using structured tables or plots.	Not applicable
Results of	20a	For each synthesis, briefly summarise the characteristics and	Page 9-20. Among the 42 included studies, parasite-based (69%), deterministic (62%), and age-structured (67%) models

S1 Table: PRISMA 2020 Checklist – Review full text and abstract

Section and Topic	Item #	Checklist item	Location where item is reported
syntheses		risk of bias among contributing studies.	predominated. Risk of bias assessment using the AMS tool classified 27 studies (62%) as very high quality and 16 (38%) as high quality; no studies were medium or low quality. Methodological gaps included limited external validation (14%) and sparse reporting of explicit timelines (33%).
	20b	Present results of all statistical syntheses conducted. If meta-analysis was done, present for each the summary estimate and its precision (e.g. confidence/credible interval) and measures of statistical heterogeneity. If comparing groups, describe the direction of the effect.	Page 9-20. No meta-analysis was performed because of substantial heterogeneity across included studies. Results were synthesized narratively and presented descriptively..
	20c	Present results of all investigations of possible causes of heterogeneity among study results.	Not applicable
	20d	Present results of all sensitivity analyses conducted to assess the robustness of the synthesized results.	Not applicable
Reporting biases	21	Present assessments of risk of bias due to missing results (arising from reporting biases) for each synthesis assessed.	Not applicable
Certainty of evidence	22	Present assessments of certainty (or confidence) in the body of evidence for each outcome assessed.	Not applicable
**DISCUSSION**			
Discussion	23a	Provide a general interpretation of the results in the context of other evidence.	Page 20. Few studies reported timelines, highlighting a key gap compared to WHO goals and prior evidence.
	23b	Discuss any limitations of the evidence included in the review.	Page 22-23. Limited reporting of time-bound predictions and underrepresentation of integrated intervention.
	23c	Discuss any limitations of the review processes used.	Page 22-23. Possible publication bias, restriction to English-language studies, and exclusion of grey literature.
	23d	Discuss implications of the results for practice, policy, and future research.	Page 22. Overall, the findings suggest that future modelling efforts may strengthen their contribution to STH control and elimination by placing greater emphasis on integrated strategies, validation-oriented forecasting, and programme-

S1 Table: PRISMA 2020 Checklist – Review full text and abstract

Section and Topic	Item #	Checklist item	Location where item is reported
			relevant decision support.
**OTHER INFORMATION**			
Registration and protocol	24a	Provide registration information for the review, including register name and registration number, or state that the review was not registered.	The review protocol attached to the manuscript
	24b	Indicate where the review protocol can be accessed, or state that a protocol was not prepared.	Not applicable
	24c	Describe and explain any amendments to information provided at registration or in the protocol.	Not applicable
Support	25	Describe sources of financial or non-financial support for the review, and the role of the funders or sponsors in the review.	Page 24. No funding
Competing interests	26	Declare any competing interests of review authors.	Page 24. Authors declare no conflict of interest
Availability of data, code and other materials	27	Report which of the following are publicly available and where they can be found: template data collection forms; data extracted from included studies; data used for all analyses; analytic code; any other materials used in the review.	Not applicable

S1 Table: PRISMA 2020 Checklist – Review full text and abstract

Section and Topic	Item #	Checklist item	Reported (Yes/No)
**TITLE**			
Title	1	Identify the report as a systematic review.	A systematic review of mathematical models for STH control: timelines and alignment with WHO goals
**BACKGROUND**			
Objectives	2	Provide an explicit statement of the main objective(s) or question(s) the review addresses.	To (i) systematically classify models based on their biological and structural characteristics; (ii) decompose STH control interventions represented in mathematical models into a set of orthogonal components and use these to develop a systematic classification framework; and (iii) synthesize model predictions in relation to WHO-defined targets and timelines.
**METHODS**			
Eligibility criteria	3	Specify the inclusion and exclusion criteria for the review.	Included studies were mathematical models of STH transmission evaluating at least one control strategy, published in English from Jan 2015 to Dec 2024.
Information sources	4	Specify the information sources (e.g. databases, registers) used to identify studies and the date when each was last searched.	Web of Science, Scopus, and Embase databases were searched for studies published between Jan 2015 and Dec 2024.
Risk of bias	5	Specify the methods used to assess risk of bias in the included studies.	The quality of included studies was assessed using the Assessment Modeling Studies Tool
Synthesis of results	6	Specify the methods used to present and synthesise results.	A narrative synthesis was used due to heterogeneity in model structures, interventions, and outcomes. Results were presented using structured tables (e.g., Tables 1–6), bar charts, pie charts, and VOSviewer-generated network/density visualizations (Figs 1–10). No meta-analysis was performed.
**RESULTS**			
Included studies	7	Give the total number of included studies and participants and summarise relevant characteristics of studies.	42 studies were included (no human participants; modeling studies). Relevant characteristics: parasite-based models (69%), deterministic frameworks (62%), age-structured populations (67%); interventions: preventive chemotherapy (81%), community-based delivery (38%), single-drug therapy (95%).

S1 Table: PRISMA 2020 Checklist – Review full text and abstract

Section and Topic	Item #	Checklist item	Reported (Yes/No)
Synthesis of results	8	Present results for main outcomes, preferably indicating the number of included studies and participants for each. If meta-analysis was done, report the summary estimate and confidence/credible interval. If comparing groups, indicate the direction of the effect (i.e. which group is favoured).	Only 33% included time-bound predictions.
**DISCUSSION**			
Limitations of evidence	9	Provide a brief summary of the limitations of the evidence included in the review (e.g. study risk of bias, inconsistency and imprecision).	Only a few studies (33%) reported time-bound outcomes, limiting insights into the duration required to achieve WHO targets.
Interpretation	10	Provide a general interpretation of the results and important implications.	The STH modeling is matured for preventive chemotherapy utilized for morbidity control but less matured in elimination question particularly in low prevalence settings where stochastic may be of more importances. Future models must standardise reporting, validate predictions, and explicitly link timelines to intervention components to support the 2030 elimination
**OTHER**			
Funding	11	Specify the primary source of funding for the review.	No external funding was received.
Registration	12	Provide the register name and registration number.	Not applicable

References

1. Page MJ, McKenzie JE, Bossuyt PM, Boutron I, Hoffmann TC, Mulrow CD, et al. The PRISMA 2020 statement: an updated guideline for reporting systematic reviews. BMJ 2021;372:n71. doi: 10.1136/bmj.n71
