# Supplementary material for: A systematic review of mathematical models for soil-transmitted helminthiasis control strategies
Source: PLoS Negl Trop Dis. 2026 Aug 28;20(8):e0013431. doi: 10.1371/journal.pntd.0013431 (PMC13537693; doi:10.1371/journal.pntd.0013431)
Supplement: Supplementary file 2 [file pntd.0013431.s002.pdf]

Table 1: S2 File: Search Strategies for All Databases

| Database | Platform | Search Strategy/Terms | Notes |
|---|---|---|---|
| Web of Science | Clarivate | ((”mathematical model*” OR ”computational model*” OR ”stochastic model*” OR ”quantitative model*” OR ”epidem* model*” OR ”theoretical model*” OR ”predictive model*” OR ”deterministic model*” OR ”individual-based model*” OR ”agent-based model*” OR ”network model*” OR ”network-based model*” OR ”compartmental model*” OR ”population model*” OR ”hybrid model*” OR ”computational approach*”) AND (”control strategy*” OR intervention* OR ”mass drug administration” OR ”preventive chemotherapy” OR sanitation OR ”hygiene promotion” OR ”deworming” OR ”public health intervention*” OR ”disease control program*” OR ”health education” OR approach* OR ”framework” OR program* OR guideline* OR ”anthelmintic treatment*” OR ”preventive intervention*” OR ”helminth control”) AND (”helminthiasis” OR ”Ascari*” OR ”roundworm” OR ”necator americanus” OR ”ancylostoma duodenale” OR ”hookworm*” OR ”Trichur*” OR ”soil-transmitted helminth*” OR ”STH” OR ”helminth infection*” OR ”intestinal parasite*” OR ”intestinal helminth*” OR ”intestinal nematode*” OR ”geohelminth*” OR ”whipworm” OR ”Strongyloides stercoralis”)) | Filters applied: Date range (2015–2024); Document type: Article; Sustainable Development Goals. |
| Embase | Elsevier | (’mathematical model’/exp OR ’mathematical model*’ OR ’computational model*’ OR ’stochastic model*’ OR ’quantitative model*’ OR ’epidem* model*’ OR ’theoretical model*’ OR ’predictive model*’ OR ’deterministic model*’ OR ’individual-based model*’ OR ’agent-based model*’ OR ’network model*’ OR ’network-based model*’ OR ’compartmental model*’ OR ’population model*’ OR ’hybrid model*’ OR ’computational approach*’) AND (’control strategy’/exp OR ’control strategy*’ OR ’intervention’/exp OR intervention* OR ’mass drug administration’/exp OR ’mass drug administration’ OR ’preventive chemotherapy’/exp OR ’preventive chemotherapy’ OR ’sanitation’/exp OR sanitation OR ’hygiene promotion’ OR ’deworming’/exp OR ’deworming’ OR ’public health intervention*’ OR ’disease control program*’ OR ’health education’/exp OR ’health education’ OR approach* OR ’framework’ OR program* OR guideline* OR ’anthelmintic treatment*’ OR ’preventive intervention*’ OR ’helminth control’) AND (’helminthiasis’/exp OR helminthiasis OR ’ascari*’ OR ’roundworm’ OR ’necator americanus’ OR ’ancylostoma duodenale’ OR ’hookworm’/exp OR ’hookworm*’ OR ’trichur*’ OR ’soil-transmitted helminth*’ OR ’sth’ OR ’helminth infection*’ OR ’intestinal parasite*’ OR ’intestinal helminth*’ OR ’intestinal nematode*’ OR ’geohelminth*’ OR ’whipworm’ OR ’strongyloides stercoralis’/exp OR ’strongyloides stercoralis’) AND [2015-2024]/py AND ’article’/it AND (’compartment model’/de OR ’mathematical model’/de OR ’predictive model’/de OR ’theoretical model’/de) | Filters applied: Publication years (2015–2024); Article type; Subject headings for mathematical and compartment models. |

| Database | Platform | Search Strategy/Terms | Notes |
|---|---|---|---|
| Scopus | Elsevier | TITLE-ABS-KEY( ("mathematical model*" OR "computational model*" OR "stochastic model*" OR "quantitative model*" OR "epidem* model*" OR "theoretical model*" OR "predictive model*" OR "deterministic model*" OR "individual-based model*" OR "agent-based model*" OR "network model*" OR "network-based model*" OR "compartmental model*" OR "population model*" OR "hybrid model*" OR "computational approach*") AND ("control strategy*" OR intervention* OR "mass drug administration" OR "preventive chemotherapy" OR sanitation OR "hygiene promotion" OR "deworming" OR "public health intervention*" OR "disease control program*" OR "health education" OR approach* OR "framework" OR program* OR guideline* OR "anthelmintic treatment*" OR "preventive intervention*" OR "helminth control") AND ("helminthiasis" OR "Ascari*" OR "roundworm" OR "necator americanus" OR "ancylostoma duodenale" OR "hookworm*" OR "Trichur*" OR "soil-transmitted helminth*" OR "STH" OR "helminth infection*" OR "intestinal parasite*" OR "intestinal helminth*" OR "intestinal nematode*" OR "geohelminth*" OR "whipworm" OR "Strongyloides stercoralis")) | Filters applied: Publication years (2015–2024); Final document type: Article. Search field: TITLE-ABS-KEY. |
