# Supplementary material for: A systematic review of mathematical models for soil-transmitted helminthiasis control strategies
Source: PLoS Negl Trop Dis. 2026 Aug 28;20(8):e0013431. doi: 10.1371/journal.pntd.0013431 (PMC13537693; doi:10.1371/journal.pntd.0013431)
Supplement: Supplementary file 3 [file pntd.0013431.s005.pdf]

A Systematic Review Protocol of Mathematical Models for Soil-Transmitted Helminthiasis Control Strategies

Lemjini Masandawa[1]*, Miracle Amadi[4], Isambi S. Mbalawata[2], Safari Kinung'hi[3], Silas S. Mirau[1]

[1] *School of Computational and Communication Science and Engineering, The Nelson Mandela African Institution of Science and Technology, P.O. Box 447, Arusha, Tanzania*

[2] *African Institute for Mathematical Sciences (AIMS) Research and Innovation Centre (RIC), NEI Global Secretariat, Rue KG590 ST, Kigali, Rwanda*

[3] *National Institute for Medical Research (NIMR), Mwanza Centre, P.O. Box 1462, Mwanza, Tanzania*

[4] *School of Engineering Science, Lappeenranta University of Technology, 53851 Lappeenranta, Finland*

1 Introduction

Soil-transmitted helminthiasis (STH) is among the diseases classified by the World Health Organization (WHO) as neglected tropical diseases (NTDs), reflecting its disproportionate burden in low-resource settings and the relatively limited research investment directed towards it [1]. STH comprises infections caused by four major nematode species: *Ascaris lumbricoides*, *Trichuris trichiura*, and the hookworms *Necator americanus* and *Ancylostoma duodenale*, with *Strongyloides stercoralis* increasingly recognized as an additional contributor to disease burden [1, 2, 3]. Globally, more than 1.5 billion people are infected across approximately 166 endemic countries, with an estimated 4.5 billion individuals at risk [4, 5, 6]. The associated burden, measured in disability-adjusted life years (DALYs), ranges from 5 to 39 million, reflecting substantial impacts on population health [7].

STH disproportionately affects impoverished and marginalized populations, particularly in regions with limited access to safe water, sanitation, and hygiene (WASH), including sub-Saharan Africa, parts of Asia, and Latin America [8]. Chronic and heavy infections are associated with anemia, malnutrition, impaired physical and cognitive development in children, and reduced productivity in adults, thereby reinforcing cycles of poverty and health inequity [9, 10].

*Corresponding author email: masandawal@nm-aist.ac.tz

Global control efforts have been guided by successive WHO roadmaps. The 2011–2020 WHO roadmap focused primarily on morbidity control through preventive chemotherapy (PC), targeting at least 75% treatment coverage among at-risk pre-school-aged children (pre-SAC) and school-aged children (SAC) [11], with subsequent expansion of treatment programmes to other at-risk groups such as women of reproductive age [12, 13]. The current 2021–2030 roadmap places greater emphasis on elimination of STH as a public health problem and on integrated approaches that combine PC with complementary measures such as WASH, health education, and other behavioural interventions [14, 15].

Preventive chemotherapy remains the cornerstone of STH control, involving the periodic administration of anthelmintic drugs such as albendazole or mebendazole to at-risk populations [4, 16]. Alternative treatment regimens have also been proposed, including dual- or triple-drug therapy, the latter defined as the combination of three anthelmintic agents, for example albendazole, ivermectin, and pyrantel pamoate [6, 5].

Mathematical models have been widely used to understand infectious disease dynamics [17, 18, 19, 20]. In the context of STH, they have become essential tools for examining transmission dynamics and evaluating intervention strategies. These models support policy decisions by simulating alternative scenarios, estimating timelines for control or elimination, and identifying optimal intervention approaches under varying epidemiological conditions [21, 22, 23].

Rationale of the study

Given the increasing reliance on mathematical models to inform STH control and elimination strategies, it is important to critically assess how this body of evidence has evolved and the extent to which it provides consistent, policy-relevant insights. Existing reviews have largely focused on broader aspects of neglected tropical disease (NTD) control [24, 25, 26, 27, 28], without specifically identifying, appraising, and synthesizing STH transmission models according to their biological and structural characteristics, the interventions represented within them, and the operational incorporation of WHO roadmap targets. Consequently, it remains unclear whether the expanding modelling evidence coherently reflects progress toward WHO targets and timelines.

A systematic review, conducted using a pre-defined protocol aligned with the PRISMA 2020 statement [29, 30], is therefore needed to address this gap and to provide a more reliable synthesis than findings drawn from individual studies.

This review aims to: (i) systematically classify STH transmission models according to their biological and structural characteristics; (ii) decompose STH control interventions represented in mathematical models into orthogonal components and classify them accordingly; and (iii) examine the operational incorporation of WHO 2011–2020 and 2021–2030 roadmap targets, together with the projected timelines reported by the included studies.

This review will contribute to the STH modelling literature by providing a structured taxonomy of mathematical models, a systematic decomposition of intervention character-

istics, an assessment of the operational incorporation of WHO roadmap targets, and a summary of methodological features (calibration, validation, sensitivity analysis, and uncertainty quantification) reported across the included literature.

The study will be guided by the following three interconnected research questions:

i. **Model taxonomy:** How are STH transmission models classified according to their biological assumptions (transmission unit), mathematical formulation, and demographic (population) structure?

ii. **Intervention taxonomy:** How are STH control interventions represented in mathematical models classified according to intervention strategy, delivery platform, target-population representation, and treatment regimen?

iii. **WHO target alignment and reported timelines:** How are WHO-defined quantitative targets or programme criteria operationally incorporated into STH model specification or analysis, and what projected timelines are reported by the included studies?

2 Materials and methods

This study will follow established procedures for conducting a systematic review in accordance with the Preferred Reporting Items for Systematic Reviews and Meta-Analyses (PRISMA) 2020 statement [29, 30]. Application of the PRISMA guidelines is intended to minimize bias at each stage of the review process and to enhance the validity of findings relating to mathematical modelling of STH control strategies. The completed PRISMA 2020 checklist and abstract will be provided as Supporting Information.

Information sources and search strategy

A comprehensive literature search will be conducted to identify peer-reviewed studies published between January 2015 and December 2024. Three electronic databases will be searched: Web of Science (Clarivate), Embase (Elsevier), and Scopus (Elsevier). Additional studies will be identified through manual screening of the reference lists of included articles and relevant review papers.

The search strategy will be structured around three core conceptual domains: (i) mathematical modelling terms, (ii) STH control intervention terms, and (iii) soil-transmitted helminth disease terms. Search terms will include controlled vocabulary (where available), free-text keywords, synonyms, and alternative spellings related to these domains. Boolean operators (AND, OR) and truncation symbols (∗) will be used to combine and expand search terms, yielding a standardized search string of the general form:

> (“mathematical model*”) AND (“soil-transmitted helminth*”) AND (“control strateg*”)

A master search strategy will be developed and tested in Web of Science and subsequently adapted to the syntax and indexing structure of Embase and Scopus. Database-specific filters will be applied to restrict results to peer-reviewed journal articles published between 2015 and 2024. The complete, database-specific search strategies will be provided in the Supporting Information.

2.1 Eligibility criteria

The search will be restricted to studies published between 2015 and 2024 to capture recent advances in mathematical modelling relevant to STH morbidity control and transmission interruption. This ten-year window ensures alignment with current global health priorities, particularly the WHO roadmaps for 2011–2020 and 2021–2030, while maintaining a manageable scope.

The inclusion criteria for this review will be:

a Mathematical models of STH transmission dynamics, including ordinary differential equation models, partial differential equation models, delay differential equation models, difference equation models, individual-based models, stochastic process models, and hybrid models combining multiple approaches.

b Models evaluating at least one STH control intervention or strategy.

c Models reporting outcomes related to infection prevalence, infection intensity, worm burden, morbidity reduction, transmission interruption, elimination thresholds, or other STH control indicators.

d Peer-reviewed journal articles published in English between January 2015 and December 2024.

The exclusion criteria will be:

a Non-dynamic models, including purely statistical analyses, regression models, or machine-learning models that do not incorporate transmission dynamics.

b Studies focusing exclusively on cost-effectiveness without incorporating transmission dynamics.

c Studies addressing only diagnostic test performance without evaluating STH transmission dynamics or control interventions.

d Models of helminth species other than STH.

e Any study that does not evaluate an STH control intervention.

f Grey literature (dissertations, reports, conference proceedings, abstracts, letters, books, and book chapters).

g Within-host models or pharmacological models that do not incorporate population-level transmission dynamics.

2.2 Study selection and consistency check

Inclusion of articles in this systematic review will proceed through three main stages: identification, screening, and eligibility assessment. Records retrieved from the three databases will be imported into EndNote by a single investigator for duplicate removal and reference management, and subsequently transferred to the Systematic Review Accelerator for additional duplicate screening. Additional records identified through reference-list screening will be added at this stage.

Screening will be conducted in two stages by five reviewers. In the first stage, titles and abstracts of all unique records will be independently screened by a subset of reviewers against the pre-defined eligibility criteria; records will be retained for full-text review if the title or abstract indicates that the criteria may be met, and excluded otherwise. In the second stage, the remaining reviewers will independently assess the full texts of retained articles for eligibility. Discrepancies at any stage will be resolved through discussion, with a third reviewer consulted where necessary. The study selection process will be reported using a PRISMA flow diagram.

Data extraction process, coding framework, and data synthesis

Data extraction will be conducted using a standardized Microsoft Excel extraction form developed for this review. Eligible studies will be distributed among the five members of the review team for independent extraction, and a random subset (approximately 10%) of extracted studies will be independently verified by a second reviewer to assess consistency. Disagreements will be resolved through discussion and, where necessary, consultation with a third reviewer.

A structured coding framework will be developed a priori, covering three domains: (i) model characteristics, (ii) intervention characteristics, and (iii) WHO target alignment and reported timelines. Classification categories will be defined a priori for the principal model and intervention dimensions, with mutually exclusive study-level categories applied where specified. Methodological characteristics and forms of WHO target incorporation will be recorded separately and may be treated as non-mutually exclusive where a study reports more than one approach or analytical role. Categories and operational definitions will be refined iteratively during the review process if additional model or intervention structures not captured by the preliminary framework are identified.

Model characteristics. Data will be extracted on three pre-specified model dimensions: transmission unit, mathematical framework, and population structure.

Transmission unit will distinguish between *host-based* models, in which infection dynamics are represented at the level of individual hosts and depletion of the susceptible population acts as the principal negative-feedback mechanism, and *parasite-based* models, in which the fundamental unit of transmission is an individual parasite or mated pair, with dynamics represented through the mean of a worm-burden distribution.

Mathematical framework will be classified as *deterministic* or *stochastic*, according to the formulation reported by the study authors. Where studies present multiple modelling approaches, the framework designated by the authors as the primary analytical approach will be used for study-level classification, while information on all reported frameworks will be extracted descriptively.

Population structure will be classified as *age-structured*, where the model explicitly incorporates age through discrete age categories or a continuous-age formulation, or *homogeneous (flat)*, where no demographic stratification is incorporated and individuals are represented as epidemiologically identical.

Classification of model characteristics will be conducted independently by the five reviewers, with disagreements resolved through discussion and consensus.

Intervention characteristics. Interventions represented in the included studies will be decomposed into four orthogonal components: intervention strategy, delivery platform, target-population representation, and treatment regimen.

Intervention strategy will be classified as preventive chemotherapy (PC), where treatment is the sole intervention evaluated, or *integrated*, where PC is combined with one or more complementary interventions such as WASH, hygiene promotion, or health education.

Delivery platform will describe the mechanism through which PC is delivered, classified as school-based delivery (SBD), community-based delivery (CBD), or combined school- and community-based delivery (SBD+CBD).

Target-population representation will describe how recipients of the primary intervention are represented in each study. Where intervention recipients are explicitly defined by demographic group, studies will be classified according to the groups receiving the intervention as WRA, SAC, PSAC+SAC, SAC+Adults, or PSAC+SAC+Adults. WRA will represent interventions targeting only women of reproductive age; SAC, interventions targeting only school-aged children; PSAC+SAC, interventions targeting both pre-school-aged and school-aged children; SAC+Adults, interventions targeting school-aged children and adults; and PSAC+SAC+Adults, interventions explicitly targeting pre-school-aged children, school-aged children, and adults. Studies applying the intervention across the modelled population without separately specifying demographic groups as intervention recipients will be classified as *community-wide*. Thus, PSAC+SAC+Adults will be distinguished from community-wide according to the representation of intervention recipients: the former will require explicit specification of PSAC, SAC, and adults, whereas the latter will apply where

intervention recipients are not separately specified by demographic group.

Treatment regimen will capture drug type (e.g., albendazole, mebendazole, or combination) and reported coverage levels (quantitative or qualitative), classified as single-drug or dual/multi-drug therapy. Where studies evaluate multiple intervention scenarios, information on all scenarios will be extracted descriptively; study-level classifications used in quantitative summaries will be based on the primary intervention scenario identified by the study authors. Missing or incompletely reported intervention characteristics will be recorded as "not reported".

WHO target alignment and reported timelines. WHO target alignment will be assessed as an indicator of policy relevance rather than model quality, recognising that mathematical models may be developed for a range of scientific, methodological, and operational purposes. The classification will assess whether and how quantitative targets or programme criteria specified in the WHO 2011–2020 and 2021–2030 roadmaps are incorporated into the model specification or analysis.

Studies will be classified as explicitly aligned when a WHO-defined quantitative target or programme criterion is operationally incorporated into the model specification or analysis. Two forms of incorporation will be distinguished according to the analytical role of the target or criterion: (i) *intervention target or input*, when a WHO quantitative programme criterion specifies the intervention level evaluated in the model; and (ii) *epidemiological target or endpoint*, when a WHO quantitative epidemiological threshold is used as the criterion against which modelled epidemiological outcomes are evaluated, including assessment of whether the specified target is attained. Incorporation in either role will be sufficient for classification as explicitly aligned. The two forms will be recorded separately and will not be mutually exclusive, as a study may use a WHO programme criterion to define an intervention scenario and also evaluate the resulting epidemiological outcome against a WHO quantitative target.

For the 2011–2020 roadmap, explicit alignment will be assigned when the WHO target of at least 75% preventive chemotherapy coverage among at-risk pre-school-aged children (PSAC) and school-aged children (SAC) is incorporated as the programme coverage target in the intervention specification or analysis. For the 2021–2030 roadmap, explicit alignment will be assigned when the WHO elimination-as-a-public-health-problem (EPHP) criterion of less than 2% moderate- and heavy-intensity (MHI) infection prevalence among PSAC and SAC, or another quantitative objective specified in the roadmap, is incorporated as an epidemiological target or endpoint.

Studies will be classified as implicitly aligned when the intervention or epidemiological outcome evaluated corresponds to a WHO roadmap objective, but the relevant WHO quantitative target or programme criterion is not operationally incorporated into the model specification or analysis. For the 2011–2020 roadmap, this will include studies evaluating morbidity control through preventive chemotherapy without using the WHO 75% coverage target as the programme coverage criterion. For the 2021–2030 roadmap, this will

include studies evaluating reductions in MHI infection or other outcomes corresponding to EPHP without applying the WHO threshold of less than 2% MHI infection prevalence as the epidemiological target or endpoint. Studies that meet neither the explicit nor implicit alignment criteria will be classified as not aligned.

Projected timelines will be coded separately from WHO target alignment. A projected timeline will be recorded when the study authors report a time interval, number of years, or projected calendar year associated with attainment of a WHO-relevant outcome. Evaluation of an outcome at a pre-specified simulation time will be classified as a projected timeline only when that time is reported as the time to attainment of the relevant outcome. Studies without a reported time to attainment will be classified as having no reported projected timeline.

WHO target alignment will be classified independently by two reviewers using these coding criteria, with disagreements resolved through consensus. Data extraction will be conducted by five reviewers, with a random subset double-extracted to assess consistency.

Given the anticipated heterogeneity in model structures, intervention types, outcome measures, and epidemiological assumptions, formal meta-analysis will be undertaken only if sufficient methodological and outcome comparability is identified. Otherwise, narrative synthesis will be used to describe patterns across the coded domains, summarise the distribution of model and intervention characteristics, and synthesise WHO roadmap alignment and reported projected timelines.

Assessment of methodological characteristics

As part of the methodological characterization of the included studies, information will be extracted on model calibration, external validation, sensitivity analysis, and uncertainty quantification, as reported by the study authors.

Calibration (parameter fitting) will be recorded where at least one model parameter is estimated or adjusted by fitting model outputs to empirical or baseline epidemiological data; the reported approach (e.g., maximum likelihood estimation, Bayesian inference, nonlinear least squares, direct fitting, or use of previously calibrated parameter sets) will be recorded, treating these categories as non-mutually exclusive.

External validation will be recorded where model predictions are compared against independent data not used for calibration, including data from a different setting, population, or time period.

Sensitivity analysis will be classified as local, global, or structural (non-mutually exclusive), and uncertainty quantification will be recorded where uncertainty in model outputs is characterized using approaches such as Bayesian posterior intervals or stochastic simulation. Sensitivity analysis and uncertainty quantification will be recorded separately, allowing studies to be classified under either or both.

Distribution of included studies by publication year. Publication year will be extracted for each included study and summarized descriptively to characterize the annual distribution of included studies over the review period.

Planned exploratory analyses. Two exploratory, post-hoc analyses are anticipated: (i) a descriptive summary of the helminth species represented across included studies, coded according to single-species or multi-species/co-infection combinations reported by the authors; and (ii) a narrative synthesis of factors (e.g., treatment intensity, drug efficacy, compliance, integrated approaches) reported by study authors in relation to projected timelines. These analyses will be considered supplementary and not primary review outcomes.

2.3 Risk of bias and assessment of study quality

The methodological quality of included studies will be assessed using an adapted version of the Assessment of Modelling Studies (AMS) framework [31, 32], developed for the appraisal of modelling studies in health and economic research. The adapted tool comprises twelve criteria: aims and objectives (A); intervention comparators (C); outcome measures (O); model structure and supporting schematic representation (S); reporting of funding sources and/or competing interests (CI); parameter specification (P); assumptions (As); data quality, sensitivity, and/or uncertainty assessment (D); model validation (V); results presentation (R); discussion and interpretation (Di); and modelling methodology (M).

Each criterion will be scored on a three-point scale (0 = absent, 1 = partially present, 2 = fully present) [33], yielding a maximum total score of 24. Total scores will be converted to percentages and categorized as low (≤50%), medium ($50\% < x \leq 65\%$), high ($65\% < x \leq 80\%$), or very high (>80%). Quality assessment will be conducted independently by the five reviewers using a standardized assessment form, with disagreements resolved through discussion and consensus. Results of the quality assessment will be summarized descriptively and considered when interpreting the review findings.

Bibliometric and network analysis

A bibliometric analysis will be conducted using VOSviewer (version 1.6.20) to characterize the research landscape of mathematical modelling studies of soil-transmitted helminthiasis. Bibliographic information will be extracted from the titles, abstracts, keywords, and author affiliations of included studies. Term co-occurrence analysis will be used to explore thematic trends, while co-authorship analysis will be used to examine patterns of scientific collaboration [34]. A minimum occurrence threshold of five will be applied for inclusion of terms in the co-occurrence network. VOSviewer's clustering algorithm will be used to group related terms and authors based on co-occurrence and co-authorship patterns, and network and density visualizations will be generated to facilitate interpretation of thematic and collaboration structures. The bibliometric analysis will be conducted as a supple-

mentary component of the review, providing descriptive insight into thematic trends and collaboration patterns within the STH modelling literature.

Handling of missing data

Missing or incompletely reported information will be recorded as reported by the original study authors. No attempt will be made to impute missing data because of the anticipated heterogeneity in model structures, intervention characteristics, and outcome measures across included studies. Missing information will be coded as "not reported" and will not constitute grounds for study exclusion. The extent and nature of missing information will be described as part of the narrative synthesis.

Anticipated outputs

The review is expected to produce: (i) a structured taxonomy of STH transmission models according to transmission unit, mathematical framework, and population structure; (ii) a decomposition and classification of STH interventions according to strategy, delivery platform, target-population representation, and treatment regimen; (iii) a descriptive account of the operational incorporation of WHO 2011–2020 and 2021–2030 roadmap targets, including explicit and implicit alignment and the analytical role of incorporated targets; (iv) a summary of projected timelines reported for WHO-relevant outcomes and associated reported determinants; (v) a methodological characterization of calibration, validation, sensitivity analysis, and uncertainty quantification practices; (vi) a quality appraisal of included studies using the adapted AMS tool; and (vii) a bibliometric characterization of thematic and collaborative structure within the literature.

Authors' contributions

Lemjini Masandawa conceived the study. Lemjini Masandawa, Safari Kinung'hi, and Isambi S. Mbalawata designed the methodology. Miracle Amadi, Silas S. Mirau, and Lemjini Masandawa will conduct data extraction and analysis. Miracle Amadi and Silas S. Mirau will perform interpretation. Lemjini Masandawa and Miracle Amadi drafted the protocol. Silas S. Mirau and Safari Kinung'hi serve as project administrators. All authors read and approved the final version of the protocol.

Funding

No external funding was received for the conduct of this study.

Declaration of competing interest

The authors declare that they have no conflict of interest.

References

[1] Neto, A. F. R., Di Christine Oliveira, Y. L., De Oliveira, L. M., La Corte, R., Jain, S., de Lyra Junior, D. P., Fujiwara, R. T., & Dolabella, S. S. (2023). Why are we still a worm world in the 2020s? An overview of risk factors and endemicity for soil-transmitted helminthiasis. *Acta Parasitologica*, 68(3), 481–495.

[2] Ng'etich, A. I., Amoah, I. D., Bux, F., & Kumari, S. (2024). Anthelmintic resistance in soil-transmitted helminths: One-Health considerations. *Parasitology Research*, 123(1), 62.

[3] Mindu, T., Chanhanga, N., Mogaka, J., & Chimbari, M. (2025). A comparative analysis of the prevalence and intensity of schistosomiasis and soil-transmitted helminth infections between preschool-aged children and school-going children in KwaZulu-Natal province. *Parasitology Research*, 124(6), 58.

[4] Okoyo, C., Medley, G., Mwandawiro, C., & Onyango, N. (2021). Modeling the interruption of the transmission of soil-transmitted helminths infections in Kenya: modeling deworming, water, and sanitation impacts. *Frontiers in Public Health*, 9, 637866.

[5] World Health Organization. (2017). *Guideline: preventive chemotherapy to control soil-transmitted helminth infections in at-risk population groups.* Geneva: World Health Organization. ISBN: 978-92-4-155011-6.

[6] World Health Organization. (2024). *Guideline: preventive chemotherapy to control soil-transmitted helminth infections in at-risk population groups.* WHO Guidelines. Licence: CC BY-NC-SA 3.0 IGO.

[7] Keiser, J., & Utzinger, J. (2008). Efficacy of current drugs against soil-transmitted helminth infections: systematic review and meta-analysis. *JAMA*, 299(16), 1937–1948.

[8] Moser, W., Schindler, C., & Keiser, J. (2017). Efficacy of recommended drugs against soil transmitted helminths: systematic review and network meta-analysis. *BMJ*, 358, j4307.

[9] Okoyo, C., Campbell, S. J., Owaga, C., Onyango, N., Medley, G., & Mwandawiro, C. (2021). Statistical regression model of water, sanitation, and hygiene; treatment coverage; and environmental influences on school-level soil-transmitted helminths and schistosome prevalence in Kenya. *The American Journal of Tropical Medicine and Hygiene*, 104(6), 2251.

[10] Le, B., Clarke, N. E., Legrand, N., & Nery, S. V. (2024). Effectiveness of ivermectin mass drug administration in the control of soil-transmitted helminth infections in endemic populations: a systematic review and meta-analysis. *Infectious Diseases of Poverty*, 13(01), 1–15.

[11] World Health Organization. (2012). *Accelerating work to overcome the global impact of neglected tropical diseases: A roadmap for implementation* (WHO/HTM/NTD/2012.5). Geneva: World Health Organization.

[12] Vegvari, C., Giardina, F., Bajaj, S., Malizia, V., Hardwick, R. J., Truscott, J. E., Montresor, A., de Vlas, S. J., Coffeng, L. E., & Anderson, R. M. (2021). Deworming women of reproductive age during adolescence and pregnancy: what is the impact on morbidity from soil-transmitted helminths infection? *Parasites & Vectors*, 14, 1–11.

[13] Coffeng, L. E., Bakker, R., Montresor, A., & de Vlas, S. J. (2015). Feasibility of controlling hookworm infection through preventive chemotherapy: a simulation study using the individual-based WORMSIM modelling framework. *Parasites & Vectors*, 8, 1–16.

[14] World Health Organization. (2020). *Ending the neglect to attain the Sustainable Development Goals: A road map for neglected tropical diseases 2021–2030* (WHO/UCN/NTD/2020.01). Geneva: World Health Organization.

[15] World Health Organization. (2020). *2030 targets for soil-transmitted helminthiases control programmes* (WHO/UCN/NTD/VVE/2020.2). Geneva: World Health Organization.

[16] Vegvari, C., Truscott, J. E., Kura, K., & Anderson, R. M. (2019). Human population movement can impede the elimination of soil-transmitted helminth transmission in regions with heterogeneity in mass drug administration coverage and transmission potential between villages: a metapopulation analysis. *Parasites & Vectors*, 12, 1–12.

[17] Masandawa, L., Mirau, S. S., & Mbalawata, I. S. (2021). Mathematical modeling of COVID-19 transmission dynamics between healthcare workers and community. *Results in Physics*, 29, 104731.

[18] Masandawa, L., Mirau, S. S., Mbalawata, I. S., Paul, J. N., Kreppel, K., & Msamba, O. M. (2022). Modeling nosocomial infection of COVID-19 transmission dynamics. *Results in Physics*, 37, 105503.

[19] Paul, J. N., Mirau, S. S., & Mbalawata, I. S. (2022). Mathematical approach to investigate stress due to control measures to curb COVID-19. *Computational and Mathematical Methods in Medicine*, 2022(1), 7772263.

[20] Paul, J. N., Mbalawata, I. S., Mirau, S. S., & Masandawa, L. (2023). Mathematical modeling of vaccination as a control measure of stress to fight COVID-19 infections. *Chaos, Solitons & Fractals*, 166, 112920.

[21] Lambura, A. G., Mwanga, G. G., Luboobi, L., & Kuznetsov, D. (2020). Mathematical model for optimal control of soil-transmitted helminth infection. *Computational and Mathematical Methods in Medicine*, 2020(1), 6721919.

[22] Oguntolu, F. A., Peter, O. J., Yusuf, A., Omede, B. I., Bolarin, G., & Ayoola, T. A. (2024). Mathematical model and analysis of the soil-transmitted helminth infections with optimal control. *Modeling Earth Systems and Environment*, 10(1), 883–897.

[23] Chazuka, Z., Chukwu, C., Mathebula, D., & Mudimu, E. (2024). Strategic approaches to mitigating hookworm infection: An optimal control and cost-effectiveness analysis. *Results in Control and Optimization*, 17, 100477.

[24] Turner, H. C., Truscott, J. E., Hollingsworth, T. D., Bettis, A. A., Brooker, S. J., & Anderson, R. M. (2015). Cost and cost-effectiveness of soil-transmitted helminth treatment programmes: systematic review and research needs. *Parasites & Vectors*, 8, 1–23.

[25] Hollingsworth, T. D., Adams, E. R., Anderson, R. M., et al. (2015). Quantitative analyses and modelling to support achievement of the 2020 goals for nine neglected tropical diseases. *Parasites & Vectors*, 8(1), 630.

[26] Montresor, A., Mupfasoni, D., Mikhailov, A., et al. (2020). The global progress of soil-transmitted helminthiases control in 2020 and World Health Organization targets for 2030. *PLoS Neglected Tropical Diseases*, 14(8), e0008505.

[27] Campbell, S. J., Nery, S. V., Doi, S. A., Gray, D. J., Soares Magalhães, R. J., McCarthy, J. S., Traub, R. J., Andrews, R. M., & Clements, A. C. (2016). Complexities and perplexities: a critical appraisal of the evidence for soil-transmitted helminth infection-related morbidity. *PLoS Neglected Tropical Diseases*, 10(5), e0004566.

[28] NTD Modelling Consortium on Soil-Transmitted Helminths. (2019). Insights from quantitative analysis and mathematical modelling on the proposed WHO 2030 goals for soil-transmitted helminths. *Gates Open Research*, 3, 1632.

[29] Page, M. J., McKenzie, J. E., Bossuyt, P. M., et al. (2021). The PRISMA 2020 statement: an updated guideline for reporting systematic reviews. *BMJ*, 372, n71.

[30] Page, M. J., McKenzie, J. E., Bossuyt, P. M., Boutron, I., Hoffmann, T. C., Mulrow, C. D., Shamseer, L., & Moher, D. (2021). Updating guidance for reporting systematic reviews: development of the PRISMA 2020 statement. *Journal of Clinical Epidemiology*, 134, 103–112.

[31] Fone, D., Hollinghurst, S., Temple, M., et al. (2003). Systematic review of the use and value of computer simulation modelling in population health and health care delivery. *Journal of Public Health*, 25(4), 325–335.

[32] Ogunlade, S. T., Meehan, M. T., Adekunle, A. I., & McBryde, E. S. (2023). A systematic review of mathematical models of dengue transmission and vector control: 2010–2020. *Viruses*, 15(1), 254.

[33] Harris, R. C., Sumner, T., Knight, G. M., & White, R. G. (2016). Systematic review of mathematical models exploring the epidemiological impact of future TB vaccines. *Human Vaccines & Immunotherapeutics*, 12(11), 2813–2832.

[34] Van Eck, N. J., & Waltman, L. (2014). Visualizing bibliometric networks. In Y. Ding, R. Rousseau, & D. Wolfram (Eds.), *Measuring Scholarly Impact: Methods and Practice* (pp. 285–320). Springer.

Supporting information

S1 File. PRISMA 2020 checklist. (PDF)

S2 File. Full database-specific search strategies. (PDF)

S3 File. Data extraction spreadsheet template. (XLSX)
